# Temporal Variation in Heat–Mortality Associations: A Multicountry Study

**DOI:** 10.1289/ehp.1409070

**Published:** 2015-05-01

**Authors:** Antonio Gasparrini, Yuming Guo, Masahiro Hashizume, Patrick L. Kinney, Elisaveta P. Petkova, Eric Lavigne, Antonella Zanobetti, Joel D. Schwartz, Aurelio Tobias, Michela Leone, Shilu Tong, Yasushi Honda, Ho Kim, Ben G. Armstrong

**Affiliations:** 1 Department of Medical Statistics, London School of Hygiene and Tropical Medicine, London, United Kingdom; 2 Division of Epidemiology and Biostatistics, School of Population Health, University of Queensland, Brisbane, Queensland, Australia; 3 Department of Pediatric Infectious Diseases, Institute of Tropical Medicine, Nagasaki University, Nagasaki, Japan; 4 Department of Environmental Health Sciences, Mailman School of Public Health, Columbia University, New York, New York, USA; 5 National Center for Disaster Preparedness, Earth Institute, Columbia University, New York, New York, USA; 6 Department of Epidemiology and Community Medicine, University of Ottawa, Ottawa, Ontario, Canada; 7 Department of Environmental Health, Harvard School of Public Health, Boston, Massachusetts, USA; 8 Institute of Environmental Assessment and Water Research (IDAEA), Spanish Council for Scientific Research (CSIC), Barcelona, Spain; 9 Department of Epidemiology, Lazio Regional Health Service, Rome, Italy; 10 School of Public Health and Social Work, Queensland University of Technology, Brisbane, Queensland, Australia; 11 Faculty of Health and Sport Sciences, University of Tsukuba, Tsukuba, Japan; 12 Graduate School of Public Health, Seoul National University, Seoul, Republic of Korea; 13 Department of Social and Environmental Health Research, London School of Hygiene and Tropical Medicine, London, United Kingdom

## Abstract

**Background:**

Recent investigations have reported a decline in the heat-related mortality risk during the last decades. However, these studies are frequently based on modeling approaches that do not fully characterize the complex temperature–mortality relationship, and are limited to single cities or countries.

**Objectives:**

We assessed the temporal variation in heat–mortality associations in a multi-country data set using flexible modelling techniques.

**Methods:**

We collected data for 272 locations in Australia, Canada, Japan, South Korea, Spain, the United Kingdom, and the United States, with a total 20,203,690 deaths occurring in summer months between 1985 and 2012. The analysis was based on two-stage time-series models. The temporal variation in heat–mortality relationships was estimated in each location with time-varying distributed lag nonlinear models, expressed through an interaction between the transformed temperature variables and time. The estimates were pooled by country through multivariate meta-analysis.

**Results:**

Mortality risk due to heat appeared to decrease over time in several countries, with relative risks associated to high temperatures significantly lower in 2006 compared with 1993 in the United States, Japan, and Spain, and a nonsignificant decrease in Canada. Temporal changes are difficult to assess in Australia and South Korea due to low statistical power, and we found little evidence of variation in the United Kingdom. In the United States, the risk seems to be completely abated in 2006 for summer temperatures below their 99th percentile, but some significant excess persists for higher temperatures in all the countries.

**Conclusions:**

We estimated a statistically significant decrease in the relative risk for heat-related mortality in 2006 compared with 1993 in the majority of countries included in the analysis.

**Citation:**

Gasparrini A, Guo Y, Hashizume M, Kinney PL, Petkova EP, Lavigne E, Zanobetti A, Schwartz JD, Tobias A, Leone M, Tong S, Honda Y, Kim H, Armstrong BG. 2015. Temporal variation in heat–mortality associations: a multicountry study. Environ Health Perspect 123:1200–1207; http://dx.doi.org/10.1289/ehp.1409070

## Introduction

High ambient temperature is an established risk factor for human health, with overwhelming evidence on the associated excess risk for mortality or morbidity outcomes (Basu 2009; Basu and Samet 2002; Ye et al. 2012). Research on the health effects of heat is usually based on time series analysis using data spanning several years or decades, and the evidence has been crucial for deriving predictions of future temperature-related health burden under climate change scenarios (Intergovernmental Panel on Climate Change 2013). Most of the studies providing estimates on the impact of climate change assume that the association between heat and health risks, as estimated from historical data, applies identically to the future (Huang et al. 2011). However, uncertainty about the actual exposure–response relationship between temperature and mortality occurring in the future is acknowledged as one of the most critical aspects for projecting the health impact in climate change studies (Linares et al. 2014; Wu et al. 2014).

Several reasons can be put forward to assume a change over time in exposure–response associations between heat and human health (Kinney et al. 2008; Patz et al. 2000). For instance, improvements in infrastructures, such as housing and air conditioning, together with socioeconomic changes and better health care and services may have decreased the susceptibility to the harmful effects of heat. Specific public health interventions may have played a role in limiting the impact, also by increasing the awareness of the health risk associated with exposure to high temperatures, and thus fostering behavioral changes or other adaptation strategies (Ebi et al. 2006).

A number of published studies have assessed this issue. Some investigations examined heat–mortality associations from single cities such as New York City, New York (Marmor 1975; Petkova et al. 2014), London (Carson et al. 2006), Stockholm, Sweden (Åström et al. 2013), and Seoul, Republic of Korea (Ha and Kim 2013), or in small countries such as the Netherlands (Ekamper et al. 2009), over long periods up to over a century. Multi-city studies conducted in the United States and Australia extended the assessment to a whole large country (Barnett 2007; Bobb et al. 2014; Coates et al. 2014; Davis et al. 2003; Guo et al. 2012; Sheridan et al. 2009). These papers provide an interesting overview of the changes and have raised hypotheses on the role of different factors that determine the susceptibility to the effect of heat. Other publications limited the analysis to more recent periods, estimating changes in the health impact of extreme temperature events in European countries (Fouillet et al. 2008; Kyselý and Kríz 2008; Kyselý and Plavcová 2012; Michelozzi et al. 2006; Morabito et al. 2012; Schifano et al. 2012). The purpose of these other assessments was to evaluate whether public health prevention programs implemented after the infamous heat wave of 2003 were successful in decreasing the health consequences of heat. We are aware of only one study that has compared changes in heat-related mortality over time in different countries (Donaldson et al. 2003).

Findings from most of these published studies suggest an attenuation in the health risks associated with high temperature over time. However, results are difficult to compare due to the adoption of alternative exposure definitions and to limitations in the analytical approaches. In this article, we contribute to the topic with an analysis of a multi-country data set. This assessment extends previous research by comparing temporal variations in heat–mortality associations from populations living in different climates and by applying flexible modeling approaches based on time-varying distributed lag nonlinear models.

## Methods

**Figure 1:**
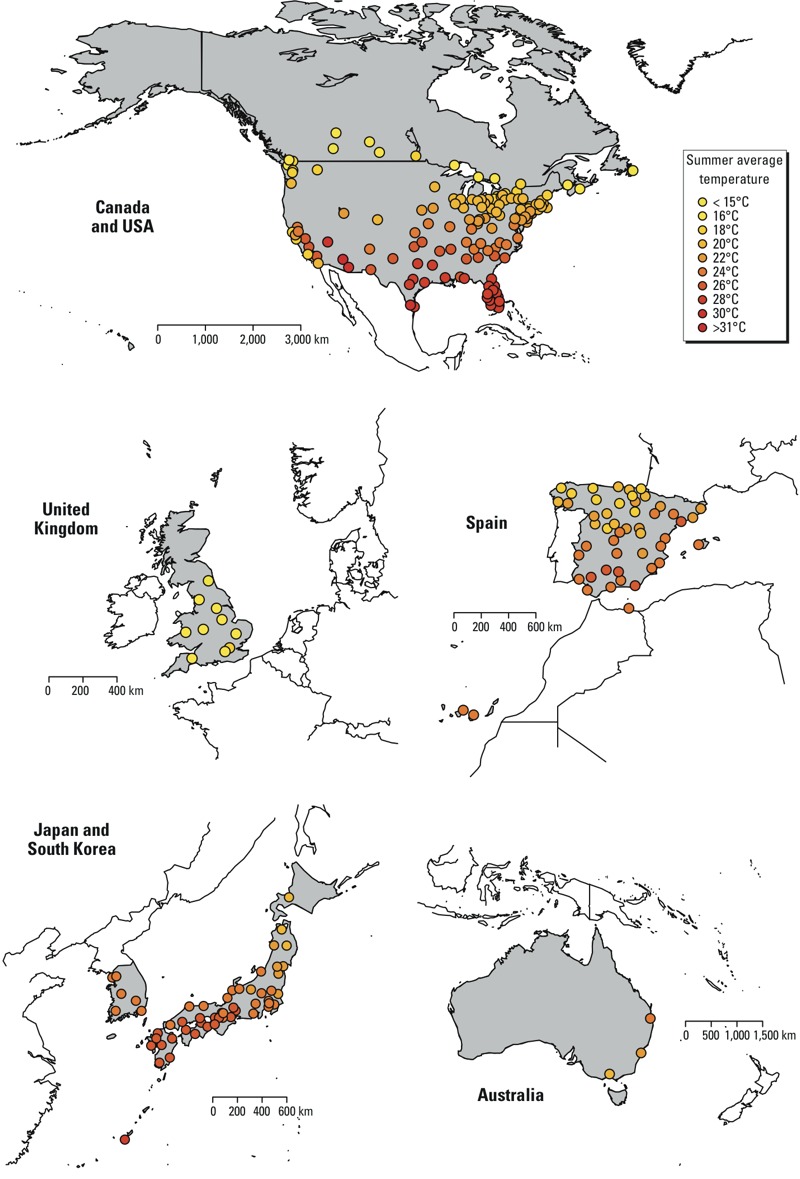
Geographic distributions of the 272 locations within the 7 countries included in the analysis, and the corresponding average mean daily temperature (°C) during the summer.

*Data*. Time-series daily data including mortality and weather variables were collected from 272 locations in seven countries: Australia (3 cities in the period 1988–2009), Canada (25 cities, 1986–2011), Japan (47 prefectures, 1985–2012), South Korea (6 cities, 1992–2010), Spain (50 cities, 1990–2010), United Kingdom (UK, 10 regions, 1993–2006), and United States (USA, 135 cities, 1985–2009). The full list of locations is provided in Supplemental Material, Table S1. These data sets include the whole population of the country (Japan, UK) or its largest metropolitan areas (Australia, Canada, South Korea, Spain, and the USA). These data have been previously used in published analysis in single-country assessments. In each location, mortality is represented by daily counts of deaths for all causes or, if not available, for nonexternal causes only [*International Classification of Diseases, 9th Revision* (ICD-9) code 0-799, and *10th Revision* (ICD-10) code A00-R99]. The exposure index was chosen as mean daily temperature, computed as the 24-hr average. We assessed this choice in sensitivity analysis, replacing the mean with maximum and minimum daily temperature. We calculated these indices either from measurements from a single monitor station closest to each metropolitan area (Canada, South Korea, Spain, and the USA) or representative of the region (Japan), or by pooling measurements from multiple monitoring stations (Australia and the UK). The data were restricted to the summer period, identified as the four warmest months of the year using average monthly temperatures. These months consistently correspond to the period December–March in Australia and to June–September in the other countries. The map shown in Figure 1 illustrates the geographical distribution of the 272 locations and the corresponding average summer temperatures. Additional details on data collection and references are provided in Supplemental Material, “Additional information about data collection.”

*First-stage time-series model*. A time-series regression for seasonal data, based on a generalized linear model with a quasi-Poisson family (Bhaskaran et al. 2013), was first applied separately in each location in order to derive estimates of location-specific temperature–mortality associations, reported as relative risk (RR). In this first-stage regression, seasonality was controlled for by using a natural cubic B-spline of day of the season with equally spaced knots and 4 degrees of freedom (df). An interaction between this spline function and indicators of summer/year was specified to relax the assumption of a constant seasonal trend. The model also includes a natural cubic B-spline of time with equally spaced knots and approximately 1 df every 10 years to control for long-term trends, and an indicator for day of the week. In the basic model, the association with temperature was specified with a standard distributed lag nonlinear model (DLNM). This class of models can describe complex nonlinear and lagged dependencies through a cross-basis function, obtained by the combination of two functions that define the conventional exposure–response relationship and the additional lag–response relationship, respectively (Gasparrini 2014). Specifically, we selected a cross-basis composed of a quadratic B-spline for the exposure response with two internal knots placed at the 50th and 90th percentiles of location-specific summer temperature distributions, and a natural cubic B-spline for the lag response with an intercept and two internal knots placed at equally spaced values in the log scale. The lag period is extended to 10 days to capture the delay in the effects of heat and to account for short-term harvesting.

*Time-varying DLNM*. The DLNMs described above assume that the bi-dimensional exposure–lag–response associations between high temperature and mortality, estimated in each location, are constant throughout the whole study period. These models are defined by 16 cross-basis variables obtained by the combination of the exposure–response and lag–response functions, with 4 df each. We extended these models to time-varying DLNMs by including a linear interaction between time and the cross-basis variables. To simplify the interpretation and make use of the standard DLNM software, we derive a special parameterization by directly defining main and interaction terms in the model. The former are represented by the cross-basis variables described above, and the latter are produced by multiplying the main terms with the time variable centred at alternative values. No main term is needed for the time variable, because this effect is already captured by the flexible spline applied to control for long-term trends. In this parameterization, the main terms represent the exposure–lag–response dependency for the day corresponding to the centering point of the time variable. Using this time-varying DLNM, we predicted the exposure–lag–response association for 2 years common to all countries (1993 and 2006) and for the first and last year of each series, by centering on the central days of the corresponding summers.

The sets of 16 coefficients of the cross-basis, estimated as constant throughout the study period and for specific years, were then reduced to sets of 4 coefficients of uni-dimensional B-splines that model the overall cumulative exposure–response relationship and the lag–response relationship at the 99th temperature percentiles (Gasparrini and Armstrong 2013). This step reduces the number of parameters to be pooled in the second-stage meta-analysis, while also preserving the complexity of the estimated dependency and thus avoiding unnecessary simplifications that can result in biases. The first-stage regression was performed with the R software (version 3.1.1), using functions in the package *dlnm* (Gasparrini 2011).

*Second-stage meta-analysis*. The estimated location-specific associations, assumed constant across the study period or predicted for specific years, were then pooled using multivariate meta-regression models of the first-stage coefficients (Gasparrini and Armstrong 2013; Gasparrini et al. 2012). Meta-regression models included indicators for country, allowing country-specific exposure–response and lag–response relationships. To account partially for the residual between-location heterogeneity attributed to different temperature distributions, we also included location-specific average temperature and temperature range as additional meta-predictors. Residual heterogeneity was tested and then quantified by the multivariate extension of the Cochran Q test and *I^2^* statistic (Gasparrini et al. 2012; Higgins and Thompson 2002).

The fitted multivariate meta-regression models were then used to derive the best linear unbiased prediction (BLUP) of the overall cumulative exposure–response associations in each location. The BLUP represents a trade-off between the location-specific relationship provided by the first-stage regression and the pooled relationship. This approach allows areas with small daily mortality counts or short series, usually characterized by very imprecise estimates, to borrow information from larger populations sharing similar characteristics (Gasparrini et al. 2012; Post et al. 2001). The second-stage meta-analysis was performed with the R package *mvmeta* (Gasparrini et al. 2012).

*Prediction and tests*. The estimates from the multivariate meta-regression can be used to obtain predictions for specific values of the meta-variables. Specifically, we derived country-specific predictions by setting the other meta-variables, average temperature and temperature range, to the average of each country. These predicted parameters, with associated (co)variance matrices, can be interpreted as country-pooled coefficients and used to reconstruct country-specific overall cumulative exposure–response relationships. Consistently with the approach adopted in the first-stage regression, with knots placed at location-specific percentiles, we represent these curves on a relative scale, along percentiles of the country-specific average summer temperature distribution. Location-specific overall cumulative exposure–response curves, represented in the original temperature range for each location, are based on BLUPs.

To ease interpretation, the curves were scaled by re-centering them on the minimum mortality percentile (MMP) of the summer temperature distribution of each country. The MMP is defined as the minimum of the exposure–response curve estimated from the model with no interaction, which can be interpreted as the average association across the whole study period. We assessed the potential temporal variation in heat–mortality associations by comparing pooled temperature–mortality curves predicted for each country in 1993 and 2006, and by comparing the curves for the first and last year included in the analysis for each country. These estimates are provided by the time-varying DLNMs with interaction terms. We also summarized the results by computing the RR at the 90th and 99th percentiles from these curves, using the MMP as the reference. Country-pooled lag–response relationships at the 99th temperature percentile were also derived from the re-centered estimates using location-specific MMPs derived from BLUPs.

Other than by visually comparing the curves, the temporal variation is assessed more formally through a statistical test. We defined a test by treating the location-specific interaction terms as cross-basis variables, reducing their coefficients and finally pooling them by country similarly to the main terms. These sets of coefficients represent the change in the overall cumulative exposure–response curves in each country. We produced a multivariate Wald test on these coefficients predicted in each country, accounting for the associated (co)variance matrix and assuming multivariate normality. The null hypothesis of the test is that none of the coefficients are different from 0, and therefore there is no change in the overall cumulative exposure–response association throughout the study period.

The R code and data to reproduce the analysis for the UK are available in a single zipped file in the Supplemental Material. An updated version is also available at the personal web page of the first author (http://www.ag-myresearch.com). The R code for the analysis on the full data is available on request. We provide details in Supplemental Material, “R code and data.”

## Results

Descriptive results are displayed in Table 1. The data set includes 20,203,690 deaths (for all causes or nonaccidental causes only) occurring during the summer within the study periods in the 272 locations of the seven countries. The table also reports the summer temperature distribution in the first half and second half of the time period included in the analysis for each country: As expected, temperature has slightly increased in some countries, although only marginally in the USA, and it has decreased in South Korea.

**Table 1:** Descriptive statistics by country: number of locations, total number of deaths, study periods (divided into first and second halves), summer temperature distribution (°C).

Country	*n*	Total deaths (*n*)	Period	Summer temperature (°C)				
				Minimum	25th	Median	75th	Maximum
Australia	3	361,135^*a*^	1988–1998	14.5	20.0	21.6	23.7	32.2
			1999–2009	14.8	20.5	22.1	24.1	32.7
Canada	25	679,693^*b*^	1986–1998	3.9	14.7	17.4	19.8	28.0
			1999–2011	4.4	15.0	17.7	20.2	28.5
Japan	47	8,117,084^*b*^	1985–1998	14.9	21.7	24.3	26.7	31.7
			1999–2012	15.3	22.6	25.1	27.3	31.6
South Korea	6	530,618^*b*^	1992–2001	14.9	21.5	23.7	26.1	31.8
			2002–2010	14.5	21.7	23.6	25.8	30.5
Spain	50	1,050,433^*b*^	1990–2000	12.4	19.7	22.2	24.3	31.1
			2001–2010	13.3	20.4	22.6	24.7	31.5
UK	10	2,285,519^*b*^	1993–1999	8.5	13.6	15.3	17.2	24.3
			2000–2006	9.3	14.3	15.9	17.7	24.6
USA	131	6,994,609^*a*^	1985–1995	11.1	21.2	23.6	25.6	31.8
			1996–2006	11.7	21.4	23.6	25.6	31.9
25th and 75th are percentiles. ^***a***^Deaths for nonaccidental causes only. ^***b***^Deaths for all causes.								

**Figure 2:**
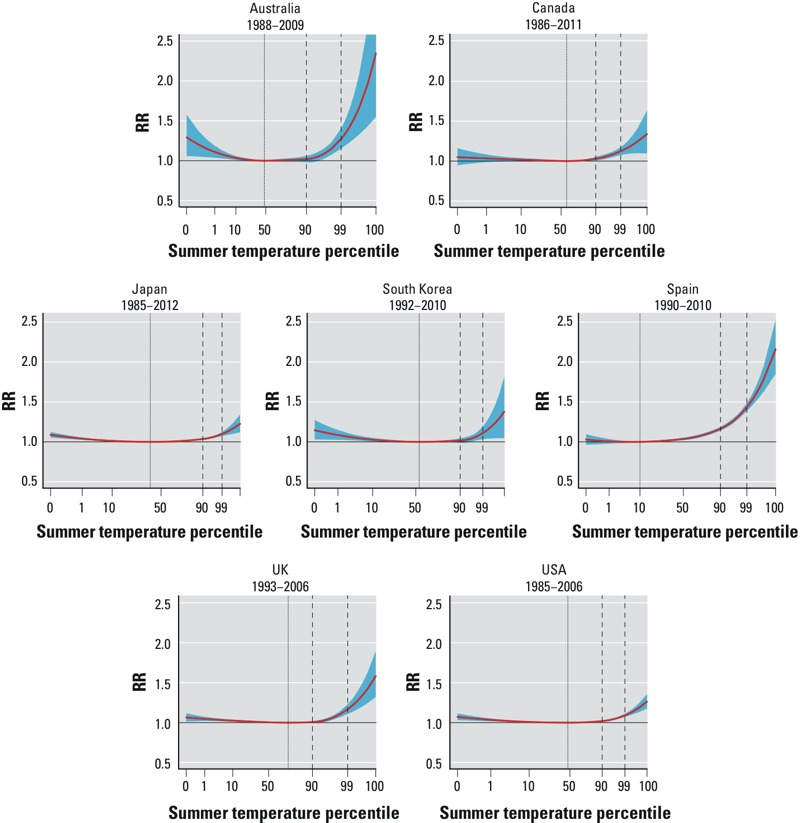
Overall cumulative exposure–response relationships*^a^* between heat and mortality predicted from the model with no interaction (interpreted as the average throughout the study period) in 7 countries, with 95% CIs. The vertical lines represent the percentile of minimum mortality temperature (dotted) and the 90th and 99th percentiles of the temperature distribution (dashed). ***^a^***The curves are represented on a relative scale of summer temperature percentiles, using country-specific distributions.

The heat–mortality associations estimated from the model with no interaction, and therefore interpretable as the average throughout the study period, are illustrated in Figure 2 and Table 2. The countries show different optimal summer temperatures, represented by MMPs ranging from the 10th percentile in Spain to 73rd percentile in the UK, as displayed in Table 2. These relationships suggest an excess mortality risk associated with high temperatures in all the countries, with the strongest estimated effects in Spain and Australia, summarized by an RR at the 99th percentile versus the MMP of 1.434 [95% confidence interval (CI): 1.386, 1.484] and 1.272 (95% CI: 1.153, 1.404), respectively. The lowest risk for the 99th percentile is estimated in the USA, with an RR of 1.091 (95% CI: 1.072, 1.110). The flexible statistical models applied here also identify an increased risk for cold summer temperatures below the MMP in almost all the countries, except for Spain.

**Table 2:** Results by country: minimum mortality percentile (MMP), period used for prediction (average, 1993, and 2006), RR for mortality (95% CI), and *p*-value of the test.

Country	MMP^*a*^	Period	RR: 90th vs. MMP	RR: 99th vs. MMP	*p*-Value^*b*^
Australia	48th	1988–2009	1.019 (0.980, 1.060)	1.272 (1.153, 1.404)	0.366
		1993	1.038 (0.986, 1.092)	1.256 (1.117, 1.412)	
		2006	0.997 (0.943, 1.055)	1.205 (1.072, 1.355)	
Canada	58th	1986–2011	1.030 (1.008, 1.051)	1.124 (1.076, 1.174)	0.125
		1993	1.043 (1.015, 1.072)	1.143 (1.084, 1.204)	
		2006	1.018 (0.993, 1.044)	1.083 (1.030, 1.138)	
Japan	40th	1985–2012	1.036 (1.026, 1.045)	1.098 (1.073, 1.124)	< 0.001
		1993	1.059 (1.047, 1.072)	1.161 (1.133, 1.190)	
		2006	1.022 (1.013, 1.031)	1.062 (1.038, 1.086)	
South Korea	54th	1992–2010	1.015 (0.987, 1.044)	1.109 (1.033, 1.191)	0.743
		1993	1.010 (0.966, 1.056)	1.094 (1.003, 1.193)	
		2006	1.021 (0.989, 1.053)	1.106 (1.010, 1.211)	
Spain	10th	1990–2010	1.165 (1.141, 1.189)	1.434 (1.386, 1.484)	< 0.001
		1993	1.191 (1.151, 1.232)	1.559 (1.478, 1.643)	
		2006	1.157 (1.128, 1.187)	1.367 (1.310, 1.426)	
UK	73rd	1993–2006	1.006 (0.993, 1.019)	1.167 (1.108, 1.230)	0.471
		1993	1.005 (0.983, 1.027)	1.158 (1.093, 1.227)	
		2006	1.014 (0.995, 1.032)	1.168 (1.111, 1.229)	
USA	47th	1985–2006	1.019 (1.012, 1.027)	1.091 (1.072, 1.110)	< 0.001
		1993	1.024 (1.016, 1.033)	1.115 (1.095, 1.136)	
		2006	0.992 (0.980, 1.004)	1.022 (0.998, 1.048)	
90th and 99th are percentiles. ^***a***^Estimated as the minimum of the overall cumulative exposure–response curve from the model without interaction (interpreted as the average across the whole study period). ^***b***^Significance test on temporal variation, based on a multivariate Wald test of the pooled reduced coefficients of the interaction terms. The null hypothesis is that no change in time occurred.					

Results from the analysis of heterogeneity are illustrated in Table 3, with a comparison of statistics from the simple multivariate random-effects meta-analysis (no meta-predictor) and multivariate random-effects meta-regressions with a single meta-predictor or all the three meta-predictors. Although the Cochran Q test provides evidence for (residual) heterogeneity in all the models, a substantial amount is actually explained by between-country differences, as indicated by the larger drop in the *I^2^* statistics when indicators for country are included as meta-predictors. The other meta-predictors—location-specific average and range of summer temperature—are borderline significant and explain a limited part of the residual heterogeneity.

**Table 3:** Second-stage random-effects meta-analysis and meta-regression models: multivariate Wald test on significance of each meta-predictor in explaining variation in overall cumulative temperature–mortality curves, Cochran Q test for heterogeneity, *I^2^* statistics for residual heterogeneity.

Model	Predictor	Test for predictor (*p*-value)	Q test (*p*-value)	*I*^2^
Intercept-only	—	—	< 0.001	54.90%
Single-predictor	Average temperature	< 0.001	< 0.001	53.10%
	Temperature range	0.057	< 0.001	54.00%
	Country	< 0.001	< 0.001	41.80%
Full	Average temperature	0.040
	Temperature range	0.026	< 0.001	38.20%
	Country	< 0.001

**Figure 3:**
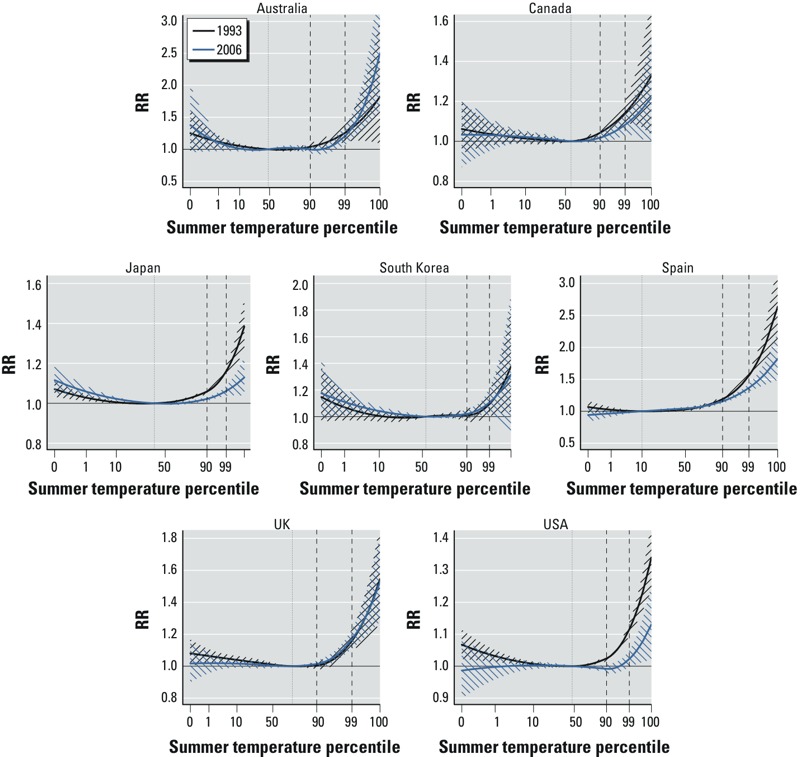
Overall cumulative exposure–response relationships*^a^* between heat and mortality predicted for 1993 (black) and 2006 (blue) in 7 countries, with 95% CIs. The vertical lines represent the percentile of minimum mortality temperature*^b^* (dotted) and the 90th and 99th percentiles of the temperature distribution (dashed). The *y*-axis is scaled to the country-specific range. ***^a^***The curves are represented on a relative scale of summer temperature percentiles, using country-specific distributions. ***^b^***Estimated as the minimum of the overall cumulative exposure–response curve from the model without interaction (interpreted as the average across the whole study period).

The main results from the analysis of the temporal variation in heat–mortality associations are summarized in Figure 3 and Table 2. Figure 3 displays the comparison of country-pooled overall cumulative exposure–response curves predicted from the time-varying DLNM for 1993 (black) and 2006 (blue) years of the series. Note that the *y*-axis is scaled to the country-specific ranges, to highlight within-country differences. Table 2 reports the overall cumulative RR at the 90th and 99th percentiles predicted for the 2 years. Corresponding results for the first and last year of the time period evaluated for each country are shown in Supplemental Material, Figure S1 and Table S2, and estimates in each of the 272 locations for 1993 and 2006 are provided in Supplemental Material, Figure S2. The analysis suggests a decrease in the mortality risk associated with heat in some of the countries. The results of the test, provided in Table 2, indicate strong evidence (*p* < 0.001) of a difference in the curves in Japan, Spain, and the USA, and some evidence (*p* = 0.125) for Canada. In particular, the USA shows the strongest relative decrease, with an RR at the 99th percentile dropping from 1.115 to 1.022. Results for Australia and South Korea are less clear, with nonsignificant tests probably attributable to the lower statistical power. There is no evidence of a change in the mortality risk associated with high temperature in the UK. A graphical representation of the effect modification of time on overall cumulative heat–mortality associations is illustrated in Supplemental Material, Figure S3. The curves can be interpreted as ratio of RR between 2006 and 1993. In particular, the significant decrease in the right tail of the curve for some countries corresponds to a significant attenuation in the RR for high temperatures in the more recent period.

The representation of nonlinear exposure–response relationships in Figure 3 provides further details on the temporal variation of the estimated effects. The analysis of the curves in in the USA suggests that the attenuation in risk can be more pronounced for less extreme temperature, with a flat region of low risk that is more extended in the last year. Specifically, the estimated RR at the 90th percentile drops from 1.024 (95% CI: 1.016, 1.033) to 0.992 (95% CI: 0.980, 1.004) in the USA (Table 2). This phenomenon does not seem to occur in Spain, where the attenuation is actually stronger for more extreme heat. However, an excess mortality risk associated with heat persists in all the countries, with RR estimated in 2006 significantly higher than 1 for temperatures higher than their 99th percentile.

**Figure 4:**
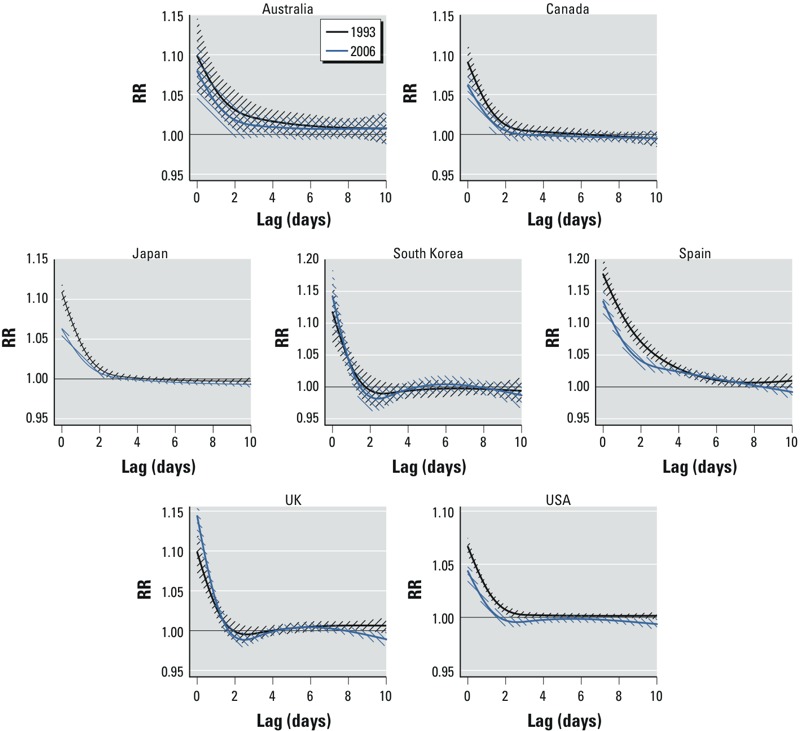
Lag–response relationships between heat and mortality predicted for 1993 (black) and 2006 (blue) in 7 countries, with 95% CIs. These curves are computed for the temperature corresponding to the 99th percentile vs. the country-specific minimum mortality temperature.*^a^* The *y*-axis is scaled to the country-specific range. ***^a^***Estimated as the minimum of the overall cumulative exposure–response curve from the model without interaction (interpreted as the average across the whole study period).

The country-pooled predictor-specific lag–response curves at the 99th summer temperature percentile for 1993 and 2006, computed versus country-specific MMPs, are represented in Figure 4. The corresponding relationships, estimated from the model without interaction and interpreted as the average throughout the study period, are provided in Supplemental Material, Figure S4. For most of the countries, most of the risk is limited to the first 2 days (lag 0–1), although Spain and Australia show a longer lag period with the risk extending up to lag 7. The graphs are consistent with the attenuation displayed in Figure 3, with a lower relative risk in 2006.

The analysis was repeated using different temperature indices, with graphs corresponding to Figure 3 for maximum and minimum daily temperatures included in Supplemental Material, Figures S5 and S6, respectively. The choice of the temperature indicator does not affect main results and interpretation.

## Discussion

This study provides evidence of attenuation in the mortality risk associated with heat in some countries during the last decades, with significant reductions in the USA, Japan, and Spain and a nonsignificant reduction in Canada. The analysis of continuous exposure–response relationships in the USA may suggest a different degree of attenuation along the temperature range, with the risk almost completely abated for values lower than the 99th percentile of summer temperature in 2006. Evidence for Australia and South Korea is unclear, with highly uncertain estimates that make it difficult to ascertain if any change occurred. In contrast, no substantial change is observed in the UK.

The results of this study allow a comparison of the effect of heat between populations experiencing different climates, and differing by socioeconomic, demographic, and other factors. Future analyses can extend the investigation to within-country differences, in particular for countries characterized by heterogeneous climatic conditions, such as the USA. The findings are generally consistent with those reported by previous investigations assessing potential changes within the same analysis periods, although a quantitative comparison is made difficult by the different definitions of the temperature–mortality relationship and by the alternative analytical approaches. Several studies have assessed temporal variations in the USA: Sheridan et al. (2009) provided evidence of a decrease in heat-related mortality rates, with a decreasing trend estimated to be between 0 and –2.6 deaths per standardized million in each decade between 1975 and 2004; Barnett (2007) estimated a decrease from 4.7% in 1987 to –0.4% in 2000 in the risk for cardiovascular mortality associated with a 10°F increase in summer temperature; Guo et al. (2012) estimated the change in the elderly mortality risk associated with an increase in temperature from the 75th percentile to the median of high temperature days in the same years, suggesting a decrease, although without reporting exact figures; Bobb et al. (2014) estimated that mortality rates associated with a 10°F increase in summer temperature attenuated from 51 deaths per 1,000 in 1987 to 19 in 2005; and Petkova et al. (2014) and colleagues estimated a decrease in the mortality RR associated with a temperature of 29°C versus 22°C of 4.6% per decade between 1973 and 2006 in New York. Estimates for other countries included in the analysis and referring to similar periods are available only for South Korea: Ha and Kim (2013) found some evidence of a decrease in heat-related cardiovascular mortality, but none for nonaccidental deaths, between the 1990s and the 2000s in Seoul.

Although the results of this study suggest that heat-related mortality risk has decreased in several countries, the drivers of such a change still need to be identified. Physiological acclimatization may have played a role in a period where extreme temperature events have become more common, even though such a strong attenuation of risk is hardly compatible with physiological changes that should have occurred in a relatively short period of time. Another reason can be the change over time of the prevalence of susceptibility factors that modify the vulnerability of a population to heat: Previous studies identified several demographic and socioeconomic characteristics as modifiers of the association (Anderson and Bell 2009; Stafoggia et al. 2006; Zanobetti et al. 2013). It is worth noting that aging of the populations across the study period would suggest an opposite change, with an increased risk in time, considering that the mortality risk due to temperature is known to rise with age. If any, this potential effect is likely to have been counterbalanced by other factors.

Infrastructural changes, such as improvements in housing and health services, could explain at least part of the estimated increase in risk. A factor previously advocated as responsible for the temporal variation is the increase in air conditioning use, as suggested by analyses of cross-sectional data (Anderson and Bell 2009; Ostro et al. 2010; Rogot et al. 1992). However, a recent study assessing the issue by comparing within-city changes in air conditioning prevalence with changes in temperature–mortality associations detected only a weak and nonsignificant effect modification (Bobb et al. 2014). Studies comparing heat–mortality relationships before and after specific weather events, such as the 2003 heat wave in Europe, suggested that the attenuation can be attributed partially to the implementation of public health intervention programs (Fouillet et al. 2008; Kyselý and Kríz 2008; Michelozzi et al. 2006; Morabito et al. 2012; Schifano et al. 2012). Beyond the direct effect of the intervention itself, such public health measures are likely to promote other changes such as behavioral adaptation and to increase awareness of the health risks associated with high temperatures. Further research is needed to disentangle the potentially composite effects of these and other factors.

The hypotheses above are consistent with the significant decrease observed in the USA, Japan, Spain, and Canada, and the lack of evidence found in the UK. The former countries have experienced a strong increase in air conditioning use within the study period, with prevalence rising on average from 32.7% to 60.4% in the USA in the years 1985–2005 (U.S. Census Bureau 2014), from 52.3% to 90.0% in Japan in the period 1985–2012 (Cabinet Office Government of Japan 2014), from 5.3% to 35.5% in Spain within 1991–2008 (Spanish Institute of National Statistics 1991, 2008), and from 28.5% to 49.4% in Canada in the years 1993–2009 (Statistics Canada (2014). In contrast, in 2011 only 3% of households had air conditioning appliances in the UK (UK Department of Energy & Climate Change 2011). In addition, although heat-surveillance systems and other public health interventions were implemented at the same time in many European countries, including Spain and the UK (Kovats and Kristie 2006; Tobias et al. 2012), the different post-2003 period of analysis (7 and 3 years, respectively) can explain why we failed to detect any change in the latter compared to the former, assuming a delay in the implementation and the potential beneficial changes of the interventions.

Alternative explanations for the potential temporal variation need to be considered. Temperature measurements are taken from monitoring stations following standardized criteria, and changes in time of these procedures to an extent that can generate substantial biases in the estimates are not expected. However, urbanization and other area-level changes may have influenced the temperature distribution near the monitoring stations, and we cannot exclude that a part of the estimated attenuation is attributable to this phenomenon.

An original contribution of this study is the application of a flexible modeling framework based on multivariate meta-analysis of reduced estimates from time-varying DLNMs. These models allow the meta-analysis of complex temperature–mortality dependencies, with exposure–response relationships measured as continuous, nonlinear shapes and accounting for effects cumulated over a lag period. The approaches proposed in most of the previous studies relied on strong approximations such as linear or linear-threshold assumptions (Barnett 2007; Bobb et al. 2014; Carson et al. 2006; Davis et al. 2003; Donaldson et al. 2003; Ekamper et al. 2009; Michelozzi et al. 2006; Morabito et al. 2012; Sheridan et al. 2009), which cannot capture the complex nonlinear associations depicted in Figures 2 and 3. In addition, the methods proposed in this contribution are able to identify different degrees of attenuation for different temperature ranges. In the USA, one of the two countries providing the highest statistical power, the increase in risk estimated in 1993 for temperatures up to the 90th percentile seems to be completely abated by 2006. In contrast, we still estimate a significant increased risk for more extreme temperatures in this year, although not as strong as the corresponding association in 1993. If confirmed in future analyses, this would indicate that the factors responsible for the attenuation might be less effective when extreme heat events occur.

The application of these flexible methods can substantially improve the characterization of temperature–health dependencies and their changes in times. The evidence can extend the knowledge on the phenomenon and inform the development of adaptation strategies to prevent or reduce the associated health impact. The modeling framework illustrated here can form the basis of future investigations on temperature–mortality associations and on the factors responsible for their temporal changes or other effect modifications.

## Conclusions

The results of this multi-country investigation provide evidence of an attenuation of the mortality impact associated with heat in the majority of countries included in the analysis, with significant reductions estimated in Japan, Spain, and the USA; a nonsignificant decrease in Canada; uncertain and nonsignificant changes in Australia and South Korea; and little evidence of any change in the UK. These findings have important implications for climate change research. In the current scenario, the application of temperature–mortality dependencies determined using historical data might overestimate the impact of high temperature in the future. Although the use of the exposure–response relationships predicted for the most recent year is likely to represent a more appropriate estimate, at least for short-term projections, the methodological framework proposed here does not provide tools to predict how the association will further change in the future. Future research must identify the drivers of temporal changes, characterizing factors and mechanisms that modify the susceptibility of a population to the health effects of heat. In addition to providing the basis for more rigorous projections of climate change impacts, this evidence will be crucial for improving public health interventions aiming at reducing the health consequences.

## Supplemental Material

(2.5 MB) PDF
