# Supplementary material for: Temporal Variation in Heat–Mortality Associations: A Multicountry Study
Source: Environ Health Perspect. 2015 May 1;123(11):1200–7. doi: 10.1289/ehp.1409070 (PMC4629745; doi:10.1289/ehp.1409070)
Supplement: (2.5 MB) PDF [file ehp.1409070.s001.acco.pdf]

Environ Health Perspect
doi: 10.1289/ehp.1409070

Note to Readers: *EHP* strives to ensure that all journal content is accessible to all readers. However, some figures and Supplemental Material published in *EHP* articles may not conform to 508 standards due to the complexity of the information being presented. If you need assistance accessing journal content, please contact ehp508@niehs.nih.gov. Our staff will work with you to assess and meet your accessibility needs within 3 working days.

Supplemental Material

Temporal Variation in Heat–Mortality Associations: A Multicountry Study

Antonio Gasparrini, Yuming Guo, Masahiro Hashizume, Patrick L. Kinney, Elisaveta P. Petkova, Eric Lavigne, Antonella Zanobetti, Joel D. Schwartz, Aurelio Tobias, Michela Leone, Shilu Tong, Yasushi Honda, Ho Kim, and Ben G. Armstrong

Table of Contents

Additional information about data collection
- Australia
- Canada
- Japan
- South Korea
- Spain
- UK
- USA

R code and data

Additional results
- **Table S1**. List of the 272 locations in 7 countries with study periods, total number of deaths and summer temperature (mean and range).
- **Table S2**. Results by country: minimum mortality percentile (MMP); period used for prediction (average, first and last year); RR for mortality (95%CI); p-value of the test.

Figure S1. Overall cumulative exposure-response relationships between heat and mortality predicted for the first (light green) and last (dark blue) year of the study period in 7 countries, with 95% confidence intervals. The vertical lines represent the minimum mortality temperature (dotted) and the 90th and 99th percentiles of the temperature distribution (dashed). Note that the *y*-axis is scaled to the country-specific range.

Figure S2. Overall cumulative exposure-response relationships between heat and mortality predicted for 1993 (light green) and 2006 (dark blue) in 272 locations, with 95% confidence intervals.

Figure S3. Effect modification of time on the overall cumulative exposure-response relationships between heat and mortality in 7 countries, expressed as ratio of RR, with 95% confidence intervals. The vertical lines represent the minimum mortality temperature (dotted) and the 90th and 99th percentiles of the temperature distribution (dashed). Note that the *y*-axis is scaled to the country-specific range.

Figure S4. Lag-response relationships between heat and mortality predicted from the model with no interaction (interpreted as the average throughout the study period) in 7 countries, with 95% confidence intervals. These curves are computed for the temperature corresponding to the 99th percentile vs the country-specific minimum mortality temperature.

Figure S5. Corresponding to Figure 1 in the manuscript with maximum daily temperature as exposure index. The vertical lines represent the minimum mortality temperature (dotted) and the 90th and 99th percentiles of the temperature distribution (dashed). Note that the *y*-axis is scaled to the country-specific range.

Figure S6. Corresponding to Figure 1 in the manuscript with minimum daily temperature as exposure index. The vertical lines represent the minimum mortality temperature (dotted) and the 90th and 99th percentiles of the temperature distribution (dashed). Note that the *y*-axis is scaled to the country-specific range.

References

Additional information about data collection

Australia

We collected data from Melbourne, Sydney and Brisbane between 1st of January 1988 and 31st of May 2009. Daily mortality, obtained from the Australian Bureau of Statistics, is represented by counts of deaths for non-external causes only (ICD-9: 0-799; ICD-10: A00-R99). Mean daily temperature (in ˚C) and relative humidity (in %), computed as the 24-hour average based on hourly measurements, were obtained from the Australian Bureau of Meteorology. We selected all available meteorological stations located within ≤30 km of each city's Central Business District (CBD) (7 stations in Brisbane, 7 stations in Melbourne and 11 stations in Sydney). We calculated the daily averages of climatic variables using all records from meteorological stations in each city. When there was a missing value (≤1.3%) for a particular meteorological station, observations recorded from the remaining weather stations were used to compute the daily average values. These data were used and described in previous publications (Tong et al. 2012; Tong et al. 2014).

Canada

We collected data from 19 census metropolitan areas (CMA) (see full list in Table S1 and in Figure S2 below) and 6 cities (Hamilton, Niagara, Oakville, Oshawa, Sarnia, Sault-Ste Marie) between 1st of January 1986 and 31st of December 2011. Daily mortality, obtained from Statistics Canada through access to the Canadian Mortality Database, is represented by counts of deaths for all causes. Mean daily temperature (in ˚C) and relative humidity (in %), computed as the 24-hour average based on hourly measurements, were obtained from Environment Canada. A single weather station was selected for each city using the airport monitoring station located closest to

the CMA centre. These data were used and described in previous publications (Kaplan et al. 2013; Martin et al. 2012).

Japan

We collected data from 47 prefectures (see full list in Table S1 and in Figure S2 below) between 1st of January 1985 and 31st of December 2012. Daily mortality, obtained from computerized death certificate data from the Ministry of Health, Labour and Welfare, Japan, is represented by counts of deaths for all causes and for non-external causes only (ICD-9: 0-799; ICD-10: A00-R99). Mean daily temperature (in ˚C) and relative humidity (in %), computed as the 24-hour average based on hourly measurements, were obtained from the Japan Meteorology Agency. A single weather station located within the urban area of the capital city was selected for each prefecture.

South Korea

We collected data from 7 cities (see full list in Table S1 and in Figure S2 below) between 1st of January 1992 and 31st of December 2010. Daily mortality is represented by counts of deaths for all causes and for non-external causes only (ICD-9: 0-799; ICD-10: A00-R99). Mean daily temperature (in ˚C) and relative humidity (in %) were computed as the 24-hour average based on hourly measurements. Daily level of pollutants were computed as the 24-hour mean based on hourly measurements.

Spain

We collected data from the 51 capital cities (see full list in Table S1 and in Figure S2 below) between 1st of January 1990 and 31st of December 2010. Daily mortality, obtained from Spain National Institute of Statistics, is represented by counts of deaths for all causes. Mean daily

temperature (in ˚C), computed as the 24-hour average based on hourly measurements, was obtained from Spain National Meteorology Agency. A single weather station, located within the urban area or at the near airport, was selected for each city. Single-day missing values were imputed as the average of the days before and after. For periods longer than two days no imputation was done. These data were used and described in previous publications (Tobias et al. 2012).

UK

We collected data in 9 regions of England and in Wales (see full list in Table S1 and in Figure S2 below) between 1st of January 1993 and 31st of December 2006. Daily mortality, obtained from the Office of National Statistics, is represented by counts of deaths for all causes and for non-external causes only (ICD-9: 0-799; ICD-10: A00-R99). Mean daily temperature (in ˚C) and relative humidity (in %), computed from the 24-h average of hourly measurements) were obtained from the British Atmospheric Data Centre. An average of 29 stations contributed data to each regional series, from a minimum of 7 in London to a maximum of 44 in Wales. Missing values were imputed through interpolation between stations within the same region. These data were used and described in previous publications (Armstrong et al. 2011; Gasparrini et al. 2012b).

USA

We collected data from 135 cities (see full list in Table S1 and in Figure S2 below) between 1st of January 1985 and 31st of December 2006. Daily mortality, obtained from the National Center for Health Statistics (NCHS), is represented by counts of deaths for non-external causes only (ICD-9: 0-799; ICD-10: A00-R99). Mean daily temperature (in ˚C, computed as the 24-hour average based on hourly measurements) and relative humidity (in %, computed from the 24-h

average of hourly measurements of dew point temperature) were obtained from the National Climatic Data Center (NCDC) of the National Oceanic and Atmospheric Administration (NOAA). A single weather station was selected for each city in the land-based station data or NCDC, based on the proximity to the city's population centre. In 6 cities where multiple observations were missing from all the nearby monitors, hourly data from the Integrated Surface Database Lite of NCDC were converted in daily values. For 25 stations missing dew point data, dew point data were obtained from the nearest station with dew point data. These data were used and described in previous publications (Zanobetti and Schwartz 2009; Zanobetti et al. 2013).

R code and data

The analysis of the multi-country data set was performed in R, version 3.1.1 (R Core Team 2014). The first and second-stage models were performed using the two packages `dlnm` (version 2.1.4) and `mvmeta` (version 0.4.5), respectively (Gasparrini 2011; Gasparrini et al. 2012a).

The R code and data reproducing all the steps of the analysis are provided in a single zipped file as Supplemental Material on the EHP website. An update version is also available at the personal web page of the first author (www.ag-myresearch.com), and at the time of writing can be accessed via the sections 'Publications' (through the link to this article) or 'R code'. The code will be updated consistently with the development of the two R packages.

The code consists of a series of scripts to be run consecutively. Although the full results cannot be reproduced as most of the data are not publicly available, we provide the data for the 10 regions of the UK used in the analysis. We anticipate that the results are similar yet not identical to those reported in the article, as the latter are based on the full data.

Additional results

Table S1. List of the 272 locations in 7 countries with study periods, total number of deaths and summer temperature (mean and range).

Location	Country	Period	Total deaths	Summer temperature (˚C)
BRISBANE	Australia	1988-2009	58836	24.2 (17.4 to 31.4)
MELBOURNE	Australia	1988-2009	140400	20.0 (10.7 to 35.0)
SYDNEY	Australia	1988-2009	161899	22.2 (14.0 to 31.8)
ABBOTSFORD	Canada	1986-2011	8572	17.0 (8.9 to 29.0)
CALGARY	Canada	1986-2011	42293	14.3 (-2.5 to 24.3)
EDMONTON	Canada	1986-2011	49262	15.3 (0.4 to 29.4)
HALIFAX	Canada	1986-2011	22407	16.9 (5.2 to 26.6)
HAMILTON	Canada	1986-2011	35884	18.9 (4.2 to 29.6)
KINGSTON	Canada	1986-2011	12362	18.8 (4.5 to 29.5)
KITCHENER-WATERLOO	Canada	1986-2011	22176	17.9 (3.6 to 29.8)
LONDON ONTARIO	Canada	1986-2011	30429	18.9 (4.3 to 30.7)
NIAGARA	Canada	1986-2011	30931	19.9 (6.2 to 30.5)
OAKVILLE	Canada	1986-2011	18543	19.4 (5.7 to 31.1)
OSHAWA	Canada	1986-2011	22707	18.3 (4.4 to 30.2)
OTTAWA	Canada	1986-2011	43017	18.7 (2.9 to 29.7)
REGINA	Canada	1986-2011	15797	16.3 (0.0 to 31.4)
SARNIA	Canada	1986-2011	8874	19.5 (6.0 to 30.7)
SUDBURY	Canada	1986-2011	13079	16.8 (1.2 to 28.8)
SAINT JOHN NB	Canada	1986-2011	13676	15.3 (4.0 to 25.7)
ST. JOHN'S NFL	Canada	1986-2011	17001	14.0 (1.2 to 24.3)
SAULT STE. MARIE	Canada	1986-2011	9327	16.4 (3.4 to 27.7)
SASKATOON	Canada	1986-2011	18102	15.8 (-1.4 to 32.1)
THUNDER BAY	Canada	1986-2011	11437	15.3 (0.9 to 27.3)
TORONTO	Canada	1986-2011	215163	19.6 (5.3 to 31.5)
VICTORIA	Canada	1986-2011	26457	15.7 (6.7 to 26.7)
VANCOUVER	Canada	1986-2011	103396	16.7 (9.2 to 28.4)
WINDSOR	Canada	1986-2011	20264	21.0 (6.0 to 31.5)
WINNIPEG	Canada	1986-2011	53136	17.2 (1.1 to 30.9)
AICHI	Japan	1985-2012	383746	25.5 (15.5 to 32.7)
AKITA	Japan	1985-2012	101267	22.0 (12.1 to 31.6)
AOMORI	Japan	1985-2012	113976	20.4 (10.6 to 30.1)
CHIBA	Japan	1985-2012	313873	24.4 (13.1 to 32.2)
EHIME	Japan	1985-2012	116503	25.6 (16.6 to 31.9)
FUKUSHIMA	Japan	1985-2012	154544	22.8 (11.3 to 31.4)
FUKUI	Japan	1985-2012	58665	24.5 (14.6 to 32.1)
FUKUOKA	Japan	1985-2012	324858	25.8 (17.5 to 32.4)

Location	Country	Period	Total deaths	Summer temperature (°C)
GIFU	Japan	1985-2012	136903	25.5 (15.6 to 32.9)
GUNMA	Japan	1985-2012	135317	24.1 (12.6 to 32.6)
HOKKAIDO	Japan	1985-2012	389479	19.6 (9.8 to 30.1)
HIROSHIMA	Japan	1985-2012	194391	25.7 (15.6 to 32.7)
HYOGO	Japan	1985-2012	354299	25.7 (16.2 to 32.0)
IBARAKI	Japan	1985-2012	189316	22.8 (11.6 to 31.3)
ISHIKAWA	Japan	1985-2012	80735	24.3 (15.0 to 32.3)
IWATE	Japan	1985-2012	107927	20.8 (10.4 to 29.3)
KAGAWA	Japan	1985-2012	76721	25.8 (16.9 to 32.3)
KANAGAWA	Japan	1985-2012	430516	24.3 (13.1 to 30.9)
KAGOSHIMA	Japan	1985-2012	146568	26.7 (18.5 to 31.1)
KOCHI	Japan	1985-2012	70061	25.6 (15.8 to 32.1)
KUMAMOTO	Japan	1985-2012	134895	26.1 (16.2 to 31.5)
KYOTO	Japan	1985-2012	170793	25.7 (15.1 to 32.8)
MIE	Japan	1985-2012	127223	25.3 (15.9 to 33.5)
MIYAGI	Japan	1985-2012	142635	21.6 (10.8 to 31.2)
MIYAZAKI	Japan	1985-2012	84538	25.8 (16.7 to 32.0)
NAGANO	Japan	1985-2012	159353	22.6 (11.9 to 30.7)
NAGASAKI	Japan	1985-2012	115970	25.7 (17.4 to 32.2)
NARA	Japan	1985-2012	87912	24.5 (14.6 to 31.7)
NIIGATA	Japan	1985-2012	184269	23.7 (14.4 to 32.6)
OITA	Japan	1985-2012	94400	25.2 (16.2 to 31.6)
OKAYAMA	Japan	1985-2012	140908	25.9 (15.9 to 32.3)
OKINAWA	Japan	1985-2012	68982	28.1 (21.1 to 31.1)
OSAKA	Japan	1985-2012	526314	26.3 (15.5 to 32.9)
SAGA	Japan	1985-2012	65842	25.8 (16.0 to 32.2)
SAITAMA	Japan	1985-2012	343311	24.4 (12.9 to 33.7)
SHIGA	Japan	1985-2012	77718	24.6 (15.1 to 31.4)
SHIMANE	Japan	1985-2012	65113	24.2 (15.0 to 32.2)
SHIZUOKA	Japan	1985-2012	234281	24.9 (14.6 to 31.9)
TOKUSHIMA	Japan	1985-2012	66168	25.6 (16.9 to 32.3)
TOCHIGI	Japan	1985-2012	130789	23.3 (12.0 to 31.4)
TOKYO	Japan	1985-2012	710556	25.0 (13.2 to 33.1)
TOTTORI	Japan	1985-2012	48663	24.5 (14.5 to 32.3)
TOYAMA	Japan	1985-2012	84518	23.9 (15.1 to 33.8)
WAKAYAMA	Japan	1985-2012	85859	25.8 (16.9 to 31.9)
YAMAGATA	Japan	1985-2012	99932	22.3 (12.0 to 31.1)
YAMAGUCHI	Japan	1985-2012	124916	25.0 (13.9 to 31.2)
YAMANASHI	Japan	1985-2012	61561	24.4 (14.7 to 31.8)
BUSAN	South Korea	1992-2010	107590	23.3 (16.1 to 30.2)
DAEGU	South Korea	1992-2010	65670	24.4 (15.5 to 32.9)

Location	Country	Period	Total deaths	Summer temperature (˚C)
DAEJEON	South Korea	1992-2010	32897	23.6 (13.0 to 31.8)
GWANGJU	South Korea	1992-2010	34231	24.2 (15.7 to 31.3)
INCHEON	South Korea	1992-2010	62066	23.0 (13.5 to 31.4)
SEOUL	South Korea	1992-2010	228164	23.7 (13.0 to 33.0)
A CORUNA	Spain	1990-2010	23135	18.9 (13.0 to 29.0)
ALBACETE	Spain	1990-2010	10495	23.0 (10.7 to 31.6)
ALICANTE	Spain	1990-2010	15634	24.7 (17.3 to 32.2)
ALMERIA	Spain	1990-2010	12709	25.2 (17.2 to 36.2)
AVILA	Spain	1990-2010	6736	19.0 (6.4 to 28.6)
BADAJOZ	Spain	1990-2010	11335	24.7 (15.1 to 33.9)
BARCELONA	Spain	1990-2010	110440	23.0 (14.2 to 30.9)
BILBAO	Spain	1990-2010	24210	19.7 (11.9 to 32.2)
BURGOS	Spain	1990-2010	13026	18.1 (6.6 to 29.8)
CACERES	Spain	1990-2010	6682	24.5 (13.6 to 34.1)
CADIZ	Spain	1990-2010	12588	23.8 (16.8 to 32.9)
CASTELLON	Spain	1990-2010	12557	24.4 (14.8 to 32.0)
CORDOBA	Spain	1990-2010	21505	26.3 (15.7 to 36.3)
CIUDAD REAL	Spain	1990-2010	6933	24.7 (11.9 to 33.7)
CUENCA	Spain	1990-2010	5159	21.6 (9.2 to 29.8)
GIRONA	Spain	1990-2010	9619	22.0 (12.3 to 30.4)
GRANADA	Spain	1990-2010	23194	24.0 (12.8 to 32.7)
GUADALAJARA	Spain	1990-2010	6275	21.5 (10.0 to 29.0)
HUELVA	Spain	1990-2010	13142	24.5 (15.8 to 36.2)
HUESCA	Spain	1990-2010	4824	22.2 (11.3 to 32.0)
JAEN	Spain	1990-2010	11011	25.2 (13.1 to 35.3)
LEON	Spain	1990-2010	13663	18.2 (7.4 to 27.9)
LLEIDA	Spain	1990-2010	11309	23.4 (12.4 to 30.6)
LOGRONO	Spain	1990-2010	10081	21.4 (9.5 to 31.5)
LUGO	Spain	1990-2010	10413	17.4 (7.9 to 29.1)
MADRID	Spain	1990-2010	171802	23.6 (10.8 to 32.4)
MALAGA	Spain	1990-2010	35549	24.8 (16.4 to 34.2)
MELILLA	Spain	1990-2010	2699	24.5 (17.5 to 36.1)
MURCIA	Spain	1990-2010	23245	24.5 (17.5 to 36.1)
OURENSE	Spain	1990-2010	11763	21.5 (10.4 to 31.5)
OVIEDO	Spain	1990-2010	21508	18.1 (10.0 to 28.4)
PAMPLONA	Spain	1990-2010	17661	20.0 (9.1 to 31.6)
PALMAS G. CANARIA	Spain	1990-2010	26984	23.8 (19.0 to 33.4)
PALMA MALLORCA	Spain	1990-2010	25347	23.6 (15.3 to 32.1)
PONTEVEDRA	Spain	1990-2010	9707	19.6 (11.6 to 30.4)
SALAMANCA	Spain	1990-2010	13985	19.8 (9.6 to 29.1)
SANTANDER	Spain	1990-2010	17796	19.3 (12.5 to 27.8)

Location	Country	Period	Total deaths	Summer temperature (°C)
TENERIFE	Spain	1990-2010	16084	24.7 (20.0 to 34.3)
SEGOVIA	Spain	1990-2010	5424	20.3 (7.5 to 31.4)
SEVILLA	Spain	1990-2010	52635	26.9 (17.0 to 36.8)
SORIA	Spain	1990-2010	4055	18.7 (7.0 to 28.1)
SAN SEBASTIAN	Spain	1990-2010	20326	18.4 (10.9 to 30.3)
TARRAGONA	Spain	1990-2010	8439	25.0 (14.2 to 32.4)
TERUEL	Spain	1990-2010	3844	20.2 (8.8 to 27.8)
TOLEDO	Spain	1990-2010	9639	24.7 (11.3 to 34.0)
VALENCIA	Spain	1990-2010	63401	24.8 (14.3 to 33.8)
VITORIA	Spain	1990-2010	11721	18.0 (7.8 to 30.6)
VALLADOLID	Spain	1990-2010	20582	20.7 (9.6 to 30.9)
ZAMORA	Spain	1990-2010	6435	21.1 (10.4 to 30.8)
ZARAGOZA	Spain	1990-2010	43127	23.7 (13.3 to 32.9)
EAST	UK	1993-2006	225749	16.3 (8.9 to 25.3)
EAST MIDLANDS	UK	1993-2006	182694	15.6 (8.5 to 24.0)
LONDON	UK	1993-2006	255713	17.5 (9.2 to 29.2)
NORTH EAST	UK	1993-2006	122923	14.6 (6.9 to 22.6)
NORTH WEST	UK	1993-2006	322820	15.4 (8.2 to 25.1)
SOUTH EAST	UK	1993-2006	342203	16.3 (8.8 to 25.3)
SOUTH WEST	UK	1993-2006	232520	15.8 (9.4 to 24.3)
WALES	UK	1993-2006	143142	15.4 (9.5 to 24.4)
WEST MIDLANDS	UK	1993-2006	232711	15.6 (8.1 to 25.2)
YORKSHIRE & HUMBER	UK	1993-2006	225044	15.3 (7.7 to 23.2)
AKRON, OH	USA	1985-2006	33295	20.3 (6.4 to 30.8)
ALBUQUERQUE, NM	USA	1985-2006	22151	23.8 (9.7 to 32.2)
ALLENTOWN-BETHLEHEM, PA	USA	1985-2006	18616	21.2 (6.9 to 31.4)
ATLANTA, GA	USA	1985-2006	96303	25.4 (11.7 to 32.5)
ATLANTIC CITY, NJ	USA	1985-2006	15199	22.1 (8.1 to 32.2)
AUSTIN, TX	USA	1985-2006	21831	28.4 (14.2 to 35.0)
BAKERSFIELD, CA	USA	1985-2006	27716	26.5 (14.2 to 36.7)
BALTIMORE, MD	USA	1985-2006	99401	23.2 (9.7 to 32.5)
BARNSTABLE-YARMOUTH, MA	USA	1985-2006	16153	19.5 (7.8 to 30.6)
BERGEN-PASSAIC, NJ	USA	1985-2006	73212	23.4 (10.6 to 34.7)
BIRMINGHAM, AL	USA	1985-2006	53165	25.6 (13.1 to 32.2)
BOSTON, MA	USA	1985-2006	145615	20.9 (7.5 to 32.2)
BATON ROUGE, LA	USA	1985-2006	19473	27.2 (14.4 to 32.8)
BROWNSVILLE, TX	USA	1985-2006	10965	28.9 (17.5 to 33.1)
BUFFALO, NY	USA	1985-2006	65843	19.8 (5.8 to 30.3)
CANTON-MASSILLON, OH	USA	1985-2006	23766	20.3 (6.4 to 30.8)
CHARLESTON, WV	USA	1985-2006	15363	22.3 (8.1 to 31.7)
CHARLOTTE, NC	USA	1985-2006	25311	24.8 (11.7 to 32.5)

Location	Country	Period	Total deaths	Summer temperature (°C)
CHATTANOOGA, TN	USA	1985-2006	18913	25.0 (12.8 to 32.8)
CHICAGO, IL	USA	1985-2006	349050	21.1 (5.6 to 33.6)
CINCINNATI, OH	USA	1985-2006	53094	22.7 (9.2 to 33.1)
CLEVELAND, OH	USA	1985-2006	125455	20.7 (7.5 to 30.8)
COLUMBIA, SC	USA	1985-2006	23635	26.2 (13.3 to 32.8)
COLUMBUS, OH	USA	1985-2006	49586	22.0 (8.3 to 31.7)
DALLAS, TX	USA	1985-2006	81635	28.1 (12.2 to 35.8)
DAYTONA BEACH, FL	USA	1985-2006	33248	27.1 (21.1 to 31.7)
DAYTON, OH	USA	1985-2006	33728	21.7 (7.2 to 32.2)
DENVER, CO	USA	1985-2006	56583	20.7 (-5.0 to 30.3)
DES MOINES, IA	USA	1985-2006	16899	22.0 (4.7 to 32.8)
DETROIT, MI	USA	1985-2006	229193	21.0 (6.4 to 31.4)
DUTCHESS COUNTY, NY	USA	1985-2006	13143	20.2 (6.4 to 31.7)
EL PASO, TX	USA	1985-2006	22366	26.8 (11.4 to 36.7)
ERIE, PA	USA	1985-2006	16916	20.3 (7.5 to 30.0)
FLINT, MI	USA	1985-2006	23674	19.6 (4.7 to 30.8)
FRESNO, CA	USA	1985-2006	31852	26.3 (13.6 to 38.6)
FT. LAUDERDALE, FL	USA	1985-2006	97450	27.4 (23.1 to 30.8)
FORT MYERS-CAPE CORAL, FL	USA	1985-2006	27339	28.2 (22.8 to 31.4)
FORT PIERCE-PORT ST. LUCIE, FL	USA	1985-2006	20681	27.3 (22.8 to 30.8)
GARY, IN	USA	1985-2006	28345	20.9 (5.3 to 31.7)
GRAND RAPIDS, MI	USA	1985-2006	24572	19.9 (4.4 to 31.7)
GREENSBORO, NC	USA	1985-2006	20318	23.7 (11.1 to 30.6)
GREENVILLE, SC	USA	1985-2006	17982	24.5 (11.7 to 32.8)
HAMILTON, OH	USA	1985-2006	15447	22.7 (9.2 to 33.1)
HARTFORD, CT	USA	1985-2006	48290	20.8 (5.8 to 31.1)
HOUSTON, TX	USA	1985-2006	115796	28.0 (15.3 to 33.3)
INDIANAPOLIS, IN	USA	1985-2006	46853	22.3 (7.8 to 32.2)
JACKSONVILLE, FL	USA	1985-2006	38724	27.0 (17.8 to 32.5)
JERSEY CITY, NJ	USA	1985-2006	31701	23.4 (10.6 to 34.7)
KANSAS CITY, MO-KS	USA	1985-2006	67807	23.5 (6.1 to 33.9)
KNOXVILLE, TN	USA	1985-2006	24834	24.1 (10.8 to 30.3)
LAKELAND-WINTER HAVEN, FL	USA	1985-2006	29250	27.9 (21.4 to 32.2)
LANCASTER, PA	USA	1985-2006	24548	21.7 (8.9 to 31.1)
LANSING, MI	USA	1985-2006	11583	19.4 (5.3 to 30.8)
LAS VEGAS, NV-AZ	USA	1985-2006	56467	30.7 (14.7 to 41.1)
LOS ANGELES, CA	USA	1985-2006	382554	20.4 (13.6 to 31.1)
LOUISVILLE, KY	USA	1985-2006	42618	24.2 (10.0 to 34.2)
LITTLE ROCK, AR	USA	1985-2006	19999	26.4 (13.3 to 35.3)
LUBBOCK, TX	USA	1985-2006	10691	25.2 (6.4 to 35.6)
MADISON, WI	USA	1985-2006	15106	19.7 (5.0 to 32.8)

Location	Country	Period	Total deaths	Summer temperature (˚C)
MCALLEN-EDINBURG-MISSION, TX	USA	1985-2006	15090	29.9 (16.7 to 36.7)
MELBOURNE-TITUSVILLE-PALM BAY, FL	USA	1985-2006	27810	27.2 (21.9 to 31.9)
MEMPHIS, TN	USA	1985-2006	47476	26.5 (13.6 to 33.9)
MIAMI, FL	USA	1985-2006	119487	28.5 (23.3 to 31.4)
MIDDLESEX, NJ	USA	1985-2006	33829	21.8 (8.6 to 32.5)
MILWAUKEE, WI	USA	1985-2006	71799	20.3 (6.1 to 33.9)
MINNEAPOLIS-ST. PAUL, MN	USA	1985-2006	74244	20.4 (4.7 to 32.5)
MOBILE, AL	USA	1985-2006	22679	26.8 (15.0 to 32.8)
MONMOUTH-OCEAN, NJ	USA	1985-2006	73356	21.8 (8.1 to 31.9)
MYRTLE BEACH, SC	USA	1985-2006	9518	25.6 (14.7 to 33.3)
NAPLES, FL	USA	1985-2006	11336	28.0 (21.9 to 30.8)
NASHUA, NH	USA	1985-2006	15701	19.4 (6.1 to 30.0)
NASHVILLE, TN	USA	1985-2006	30097	24.9 (11.1 to 32.5)
NASSAU-SUFFOLK, NY	USA	1985-2006	143694	21.4 (8.6 to 32.2)
NEWARK, NJ	USA	1985-2006	68728	23.4 (10.6 to 34.7)
NEWBURGH, NY	USA	1985-2006	15476	20.6 (6.7 to 30.8)
NEW HAVEN-MERIDEN, CT	USA	1985-2006	48273	20.8 (5.8 to 31.1)
NEW LONDON, CT	USA	1985-2006	12576	20.7 (7.8 to 31.1)
NEW YORK, NY	USA	1985-2006	426430	23.4 (10.3 to 34.4)
OAKLAND, CA	USA	1985-2006	99888	18.1 (12.2 to 27.5)
OCALA, FL	USA	1985-2006	17933	27.2 (19.4 to 32.2)
OKLAHOMA CITY, OK	USA	1985-2006	36601	25.7 (7.5 to 34.7)
OMAHA, NE	USA	1985-2006	22024	22.3 (2.2 to 33.3)
ORANGE COUNTY, CA	USA	1985-2006	98795	22.4 (14.4 to 32.8)
ORLANDO, FL	USA	1985-2006	49272	27.8 (21.9 to 32.2)
PENSACOLA, FL	USA	1985-2006	16165	27.3 (16.9 to 34.2)
PHILADELPHIA, PA-NJ	USA	1985-2006	279833	23.5 (9.4 to 33.3)
PHOENIX, AZ	USA	1985-2006	117774	33.2 (18.9 to 41.4)
PITTSBURGH, PA	USA	1985-2006	97477	20.9 (7.8 to 30.3)
PORTLAND, ME	USA	1985-2006	14093	18.5 (5.0 to 29.7)
PORTLAND, OR	USA	1985-2006	65670	19.4 (10.6 to 29.7)
PROVIDENCE-FALL RIVER, RI-MA	USA	1985-2006	11215	20.9 (7.2 to 31.4)
PUNTA GORDA, FL	USA	1985-2006	11663	28.0 (23.1 to 32.2)
RALEIGH, NC	USA	1985-2006	18149	24.5 (12.5 to 32.2)
READING, PA	USA	1985-2006	22163	24.5 (12.5 to 32.2)
RIVERSIDE-SAN BERNARDINO, CA	USA	1985-2006	134247	25.1 (12.2 to 36.7)
ROCHESTER, NY	USA	1985-2006	39921	19.6 (5.0 to 30.3)
ROCKFORD, IL	USA	1985-2006	14176	20.7 (4.7 to 31.7)
SACRAMENTO, CA	USA	1985-2006	53242	23.1 (13.3 to 34.7)
SAGINAW, MI	USA	1985-2006	12524	19.6 (5.3 to 31.1)

Location	Country	Period	Total deaths	Summer temperature (˚C)
SALINAS, CA	USA	1985-2006	14338	17.3 (12.2 to 25.8)
SALT LAKE CITY, UT	USA	1985-2006	27783	22.8 (5.6 to 32.8)
SAN ANTONIO, TX	USA	1985-2006	57736	28.4 (12.8 to 34.2)
SARASOTA-BRADENTON, FL	USA	1985-2006	46351	28.0 (20.6 to 32.8)
SCRANTON--WILKES-BARRE--HAZLETON, PA	USA	1985-2006	46123	20.0 (5.8 to 31.9)
SAN DIEGO, CA	USA	1985-2006	115049	20.9 (14.7 to 30.8)
SEATTLE, WA	USA	1985-2006	69921	17.5 (8.9 to 28.1)
SAN FRANCISCO, CA	USA	1985-2006	77042	17.6 (11.9 to 30.0)
SHREVEPORT, LA	USA	1985-2006	16221	27.2 (13.6 to 34.2)
SAN JOSE, CA	USA	1985-2006	54245	21.0 (13.9 to 32.2)
SPOKANE, WA	USA	1985-2006	21294	18.3 (4.4 to 30.6)
SPRINGFIELD, MA	USA	1985-2006	29030	20.8 (5.8 to 31.1)
STAMFORD-NORWALK, CT	USA	1985-2006	43691	21.5 (8.6 to 30.8)
ST. LOUIS, MO-IL	USA	1985-2006	96368	24.6 (9.2 to 33.6)
STOCKTON-LODI, CA	USA	1985-2006	25244	23.8 (13.9 to 36.9)
SYRACUSE, NY	USA	1985-2006	26252	19.8 (5.0 to 30.3)
TACOMA, WA	USA	1985-2006	29688	17.8 (10.0 to 26.7)
TAMPA-ST. PETERSBURG-CLEARWATER, FL	USA	1985-2006	49811	28.0 (21.7 to 31.7)
TOLEDO, OH	USA	1985-2006	28669	20.9 (6.9 to 31.7)
TRENTON, NJ	USA	1985-2006	17869	21.6 (7.8 to 31.7)
TUCSON, AZ	USA	1985-2006	39643	29.5 (18.6 to 37.2)
TULSA, OK	USA	1985-2006	29746	26.1 (8.6 to 35.3)
UTICA-ROME, NY	USA	1985-2006	16479	19.0 (4.4 to 28.9)
VENTURA COUNTY, CA	USA	1985-2006	27134	18.9 (13.1 to 26.4)
VIRGINIA BEACH, VA	USA	1985-2006	58165	24.9 (13.9 to 33.1)
WASHINGTON, DC-MD-VA	USA	1985-2006	44237	24.4 (10.6 to 33.9)
WICHITA, KS	USA	1985-2006	21215	24.9 (5.0 to 33.9)
WILMINGTON, DE	USA	1985-2006	23455	22.8 (9.2 to 31.9)
WORCESTER, MA	USA	1985-2006	41347	19.0 (3.3 to 28.9)
WEST PALM BEACH-BOCA RATON, FL	USA	1985-2006	72824	28.0 (23.3 to 31.1)
YORK, PA	USA	1985-2006	19280	22.0 (8.3 to 31.1)
YOUNGSTOWN-WARREN, OH	USA	1985-2006	26632	19.6 (6.4 to 30.6)

Table S2. Results by country: minimum mortality percentile (MMP); period used for prediction (average, first and last year); RR for mortality (95%CI); p-value of the test.

	MMP	Period	RR: 90th vs MMP	RR: 99th vs MMP	p-value[a]
		1988-2009	1.019 (0.980, 1.060)	1.272 (1.153, 1.404)	
Australia	48th	1988	1.049 (0.972, 1.132)	1.271 (1.077, 1.499)	0.374
		2006	0.993 (0.927, 1.064)	1.198 (1.048, 1.370)	
		1986-2011	1.030 (1.008, 1.051)	1.124 (1.076, 1.174)	
Canada	58th	1986	1.049 (1.007, 1.094)	1.188 (1.099, 1.283)	0.119
		2011	1.011 (0.975, 1.049)	1.066 (1.000, 1.136)	
		1985-2012	1.036 (1.026, 1.045)	1.098 (1.073, 1.124)	
Japan	40nd	1985	1.083 (1.066, 1.101)	1.227 (1.186, 1.271)	<0.001
		2012	1.006 (0.993, 1.019)	1.024 (0.997, 1.052)	
		1992-2010	1.015 (0.987, 1.044)	1.109 (1.033, 1.191)	
South Korea	54th	1992	1.011 (0.962, 1.063)	1.090 (0.986, 1.205)	0.761
		2010	1.025 (0.979, 1.074)	1.107 (0.990, 1.239)	
		1990-2010	1.165 (1.141, 1.189)	1.434 (1.386, 1.484)	
Spain	10th	1990	1.198 (1.149, 1.249)	1.613 (1.509, 1.723)	<0.001
		2010	1.156 (1.115, 1.198)	1.292 (1.219, 1.369)	
		1993-2006	1.006 (0.993, 1.019)	1.167 (1.108, 1.230)	
UK	73th	1993	1.004 (0.981, 1.028)	1.161 (1.088, 1.237)	0.477
		2006	1.014 (0.994, 1.034)	1.169 (1.112, 1.229)	
		1985-2006	1.019 (1.012, 1.027)	1.091 (1.072, 1.110)	
USA	47nd	1985	1.047 (1.032, 1.062)	1.180 (1.147, 1.215)	<0.001
		2006	0.993 (0.980, 1.005)	1.023 (0.999, 1.049)	

[a]Significance test of the effect modification, based on a multivariate Wald test of the pooled reduced coefficients of the interaction terms. The null hypothesis is that no change in time occurred.

Figure S1. Overall cumulative exposure-response relationships[a] between heat and mortality predicted for the first (light green) and last (dark blue) year of the study period in 7 countries, with 95% confidence intervals. The vertical lines represent the minimum mortality temperature[b] (dotted) and the 90th and 99th percentiles of the temperature distribution (dashed). Note that the *y*-axis is scaled to the country-specific range. [a]The curves are represented on a relative scale of summer temperature percentiles, using country-specific distributions. [b]Estimated as the minimum of the overall cumulative exposure-response curve from the model without interaction (interpreted as the average across the whole study period).

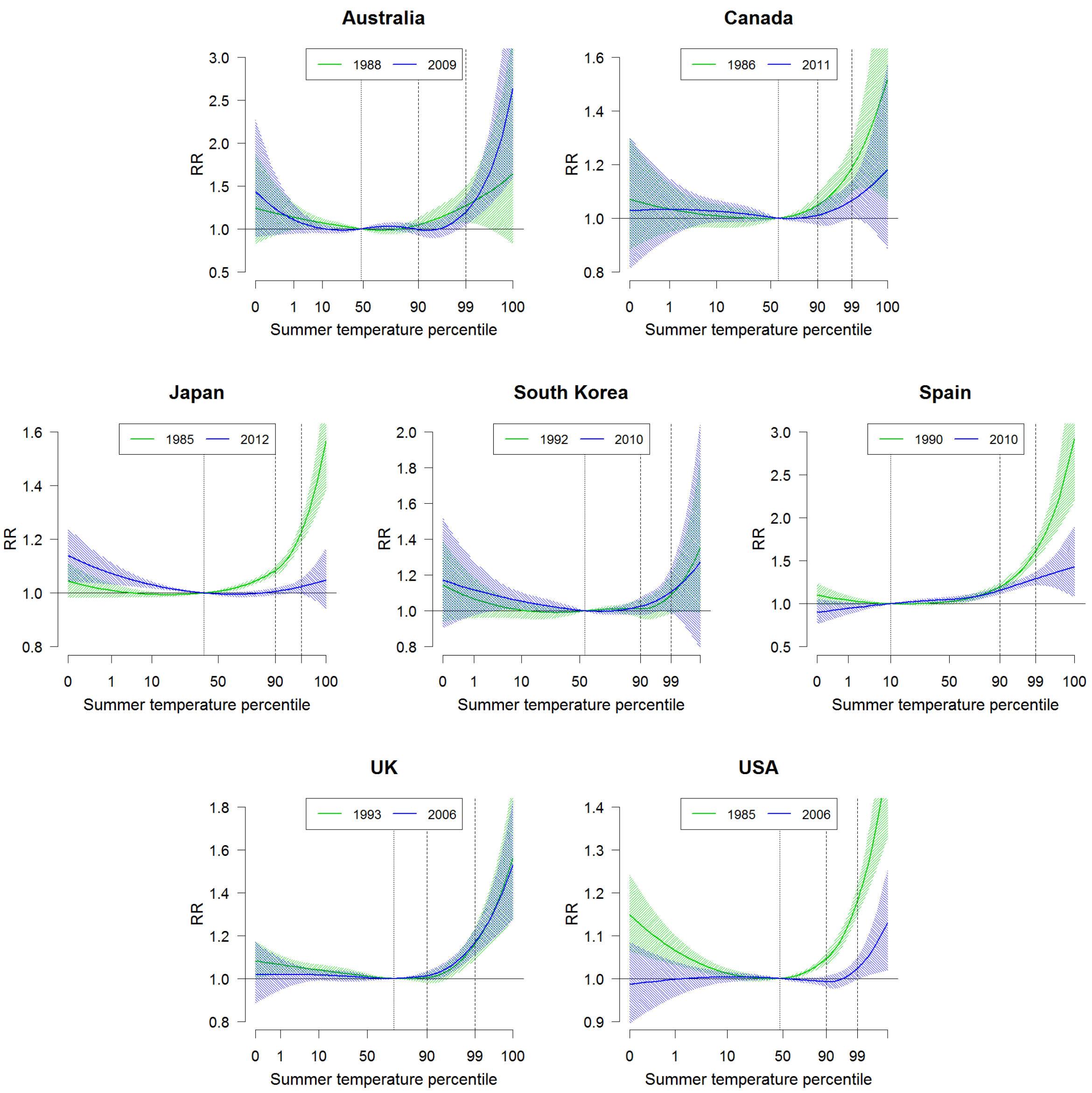

Figure S2. Overall cumulative exposure-response relationships[a] between heat and mortality predicted for 1993 (light green) and 2006 (dark blue) in 272 locations, with 95% confidence intervals. [a]The curves are represented on a relative scale of summer temperature percentiles, using country-specific distributions.

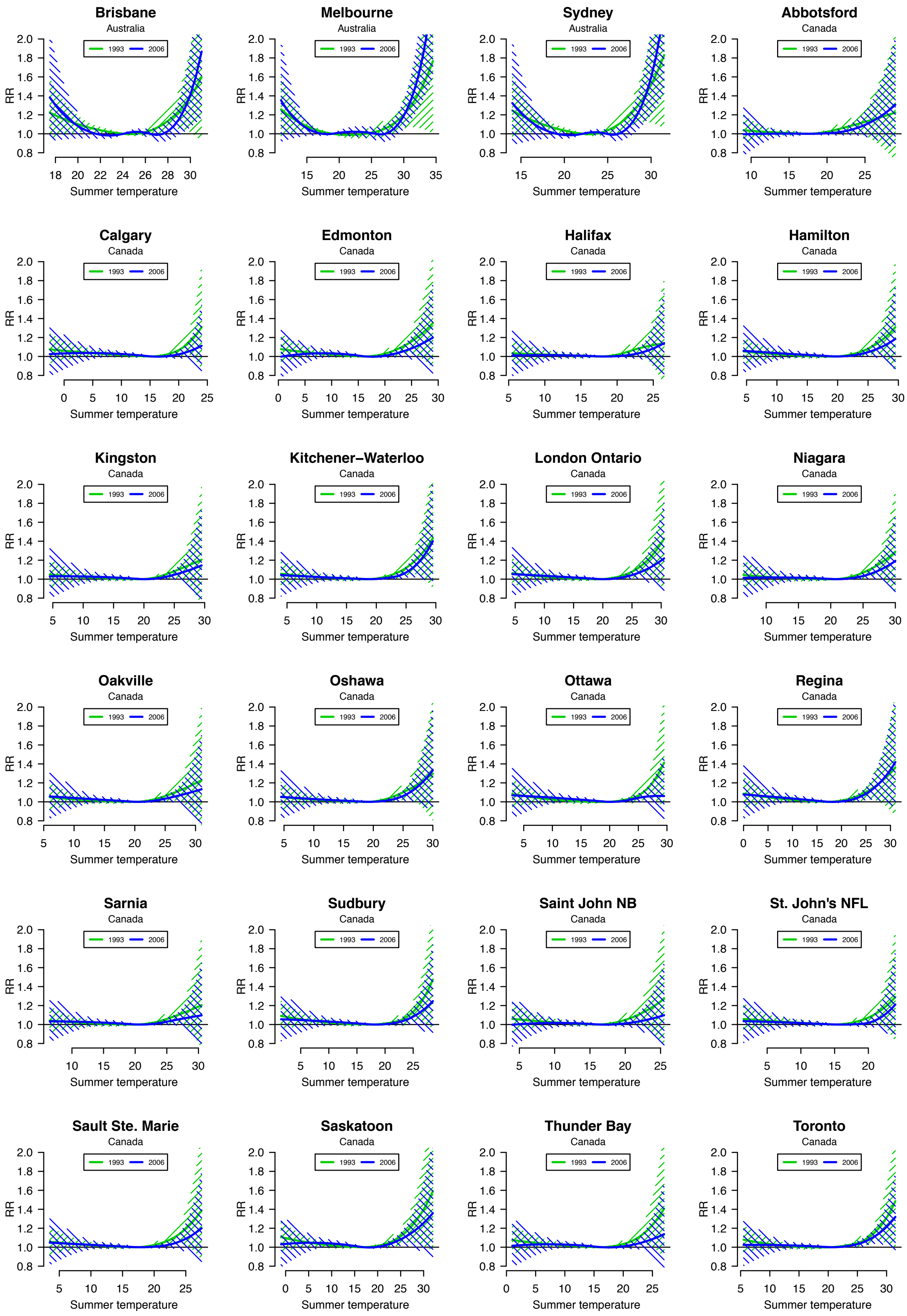
Brisbane
Australia
Melbourne
Australia
Sydney
Australia
Abbotsford
Canada
Calgary
Canada
Edmonton
Canada
Halifax
Canada
Hamilton
Canada
Kingston
Canada
Kitchener–Waterloo
Canada
London Ontario
Canada
Niagara
Canada
Oakville
Canada
Oshawa
Canada
Ottawa
Canada
Regina
Canada
Sarnia
Canada
Sudbury
Canada
Saint John NB
Canada
St. John's NFL
Canada
Sault Ste. Marie
Canada
Saskatoon
Canada
Thunder Bay
Canada
Toronto
Canada
1993
2006
RR
Summer temperature

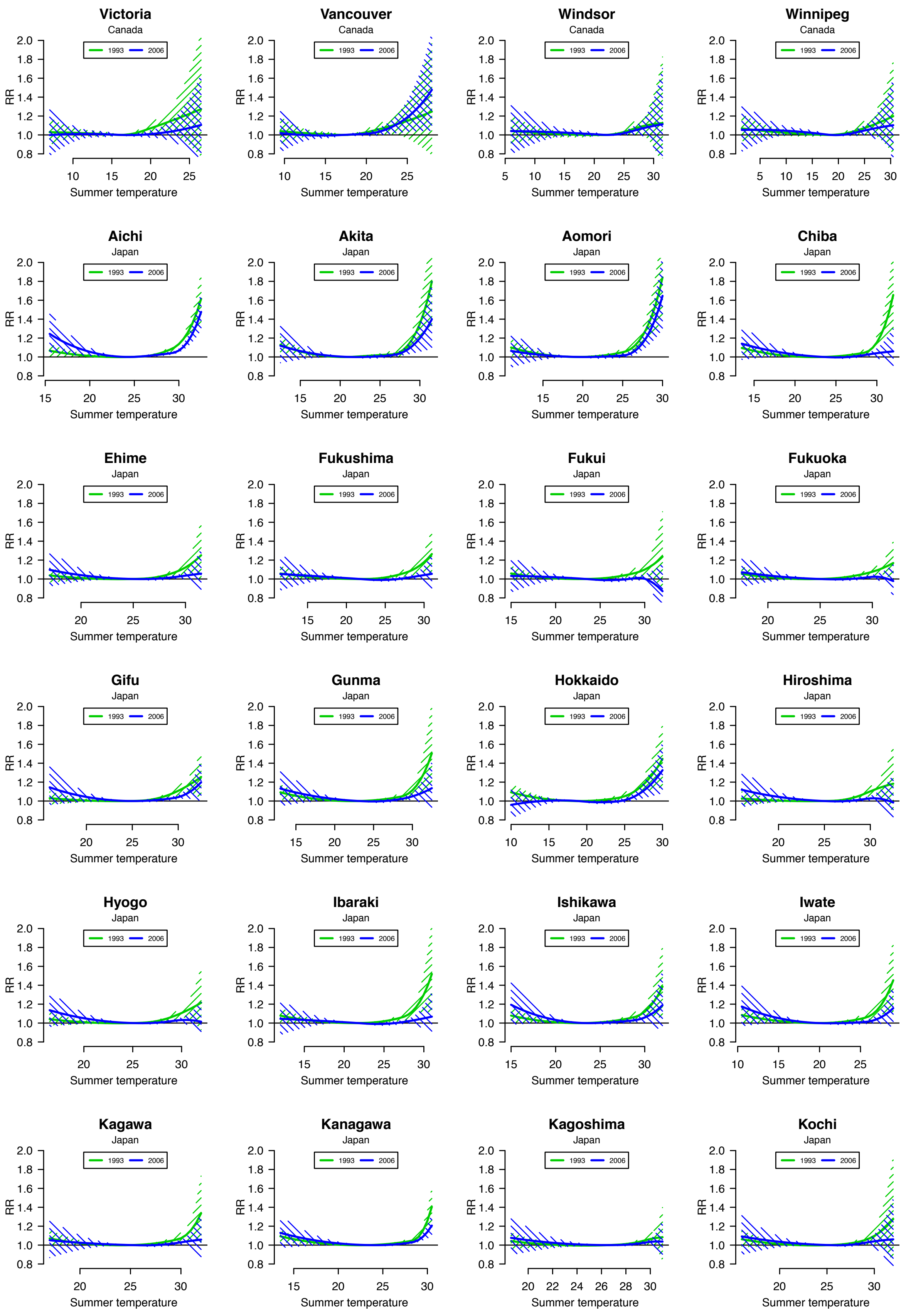
Victoria
Canada
1993 2006
RR
Summer temperature
Vancouver
Canada
Windsor
Canada
Winnipeg
Canada
Aichi
Japan
Akita
Japan
Aomori
Japan
Chiba
Japan
Ehime
Japan
Fukushima
Japan
Fukui
Japan
Fukuoka
Japan
Gifu
Japan
Gunma
Japan
Hokkaido
Japan
Hiroshima
Japan
Hyogo
Japan
Ibaraki
Japan
Ishikawa
Japan
Iwate
Japan
Kagawa
Japan
Kanagawa
Japan
Kagoshima
Japan
Kochi
Japan

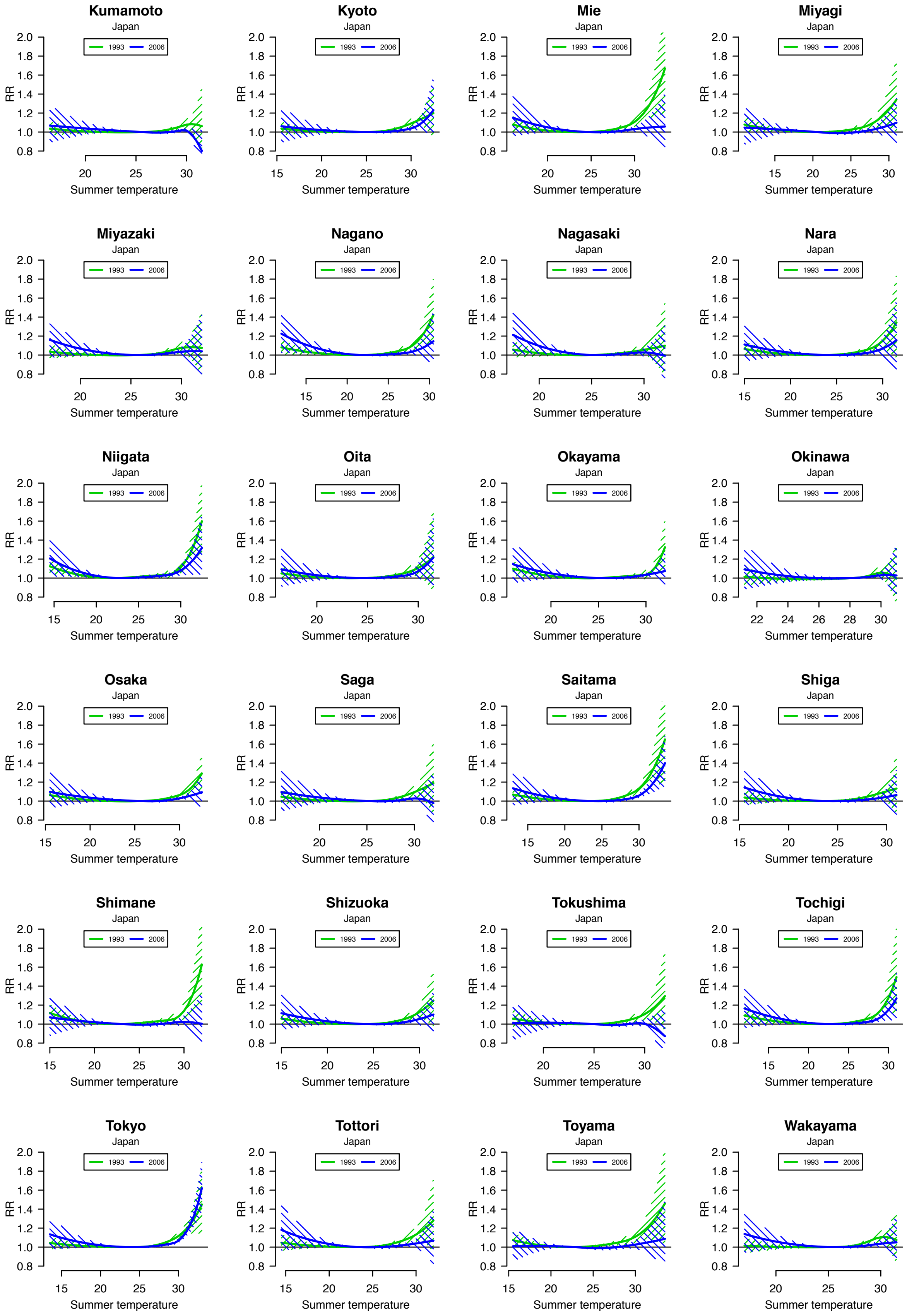

Kumamoto
Kyoto
Mie
Miyagi
Miyazaki
Nagano
Nagasaki
Nara
Niigata
Oita
Okayama
Okinawa
Osaka
Saga
Saitama
Shiga
Shimane
Shizuoka
Tokushima
Tochigi
Tokyo
Tottori
Toyama
Wakayama
Japan
1993
2006
RR
Summer temperature

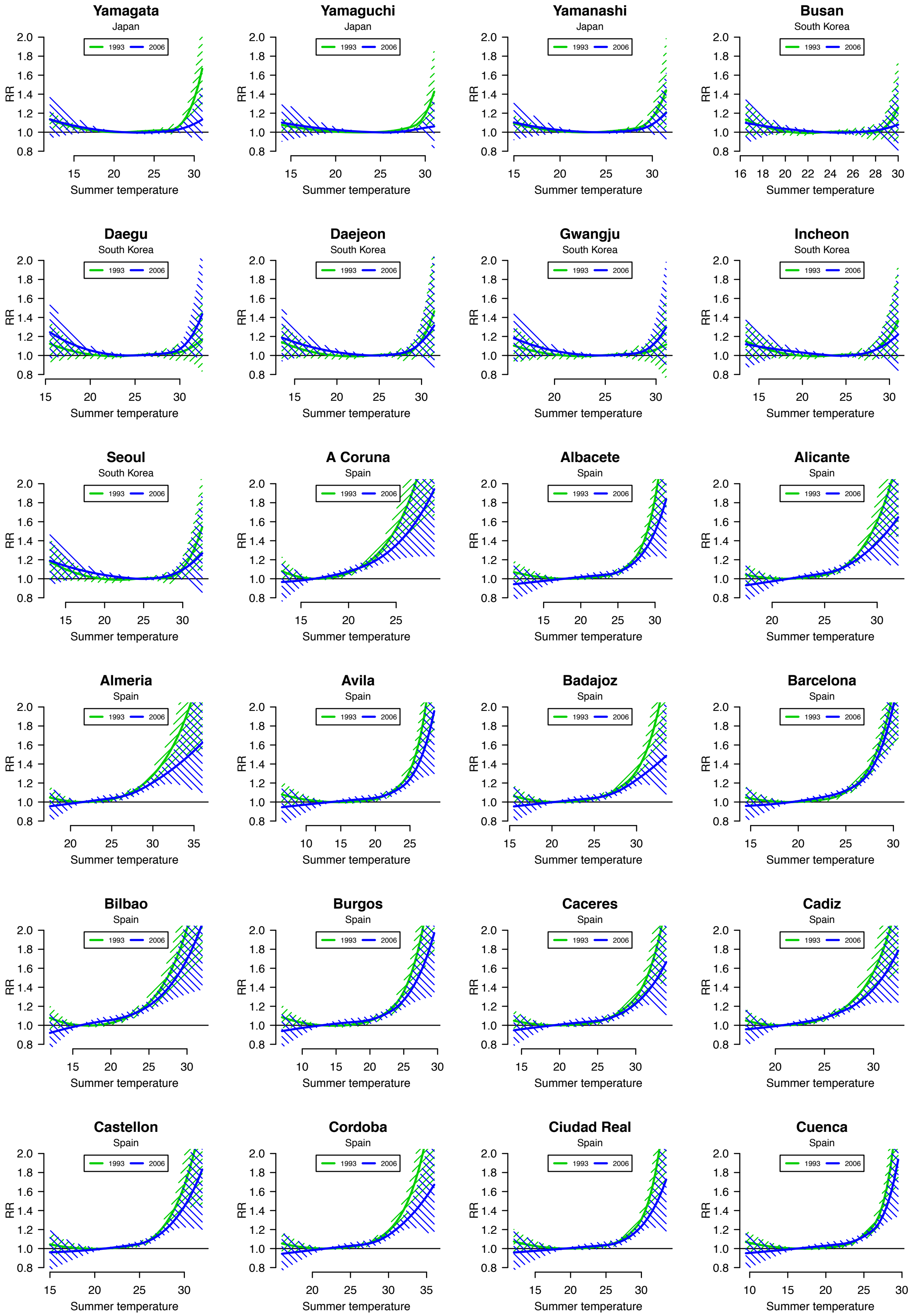
Yamagata
Japan
1993 2006
RR
2.0 1.8 1.6 1.4 1.2 1.0 0.8
15 20 25 30
Summer temperature
Yamaguchi
Japan
1993 2006
RR
15 20 25 30
Summer temperature
Yamanashi
Japan
1993 2006
RR
15 20 25 30
Summer temperature
Busan
South Korea
1993 2006
RR
16 18 20 22 24 26 28 30
Summer temperature
Daegu
South Korea
1993 2006
RR
15 20 25 30
Summer temperature
Daejeon
South Korea
1993 2006
RR
15 20 25 30
Summer temperature
Gwangju
South Korea
1993 2006
RR
20 25 30
Summer temperature
Incheon
South Korea
1993 2006
RR
15 20 25 30
Summer temperature
Seoul
South Korea
1993 2006
RR
15 20 25 30
Summer temperature
A Coruna
Spain
1993 2006
RR
15 20 25
Summer temperature
Albacete
Spain
1993 2006
RR
15 20 25 30
Summer temperature
Alicante
Spain
1993 2006
RR
20 25 30
Summer temperature
Almeria
Spain
1993 2006
RR
20 25 30 35
Summer temperature
Avila
Spain
1993 2006
RR
10 15 20 25
Summer temperature
Badajoz
Spain
1993 2006
RR
15 20 25 30
Summer temperature
Barcelona
Spain
1993 2006
RR
15 20 25 30
Summer temperature
Bilbao
Spain
1993 2006
RR
15 20 25 30
Summer temperature
Burgos
Spain
1993 2006
RR
10 15 20 25 30
Summer temperature
Caceres
Spain
1993 2006
RR
15 20 25 30
Summer temperature
Cadiz
Spain
1993 2006
RR
20 25 30
Summer temperature
Castellon
Spain
1993 2006
RR
15 20 25 30
Summer temperature
Cordoba
Spain
1993 2006
RR
20 25 30 35
Summer temperature
Ciudad Real
Spain
1993 2006
RR
15 20 25 30
Summer temperature
Cuenca
Spain
1993 2006
RR
10 15 20 25 30
Summer temperature

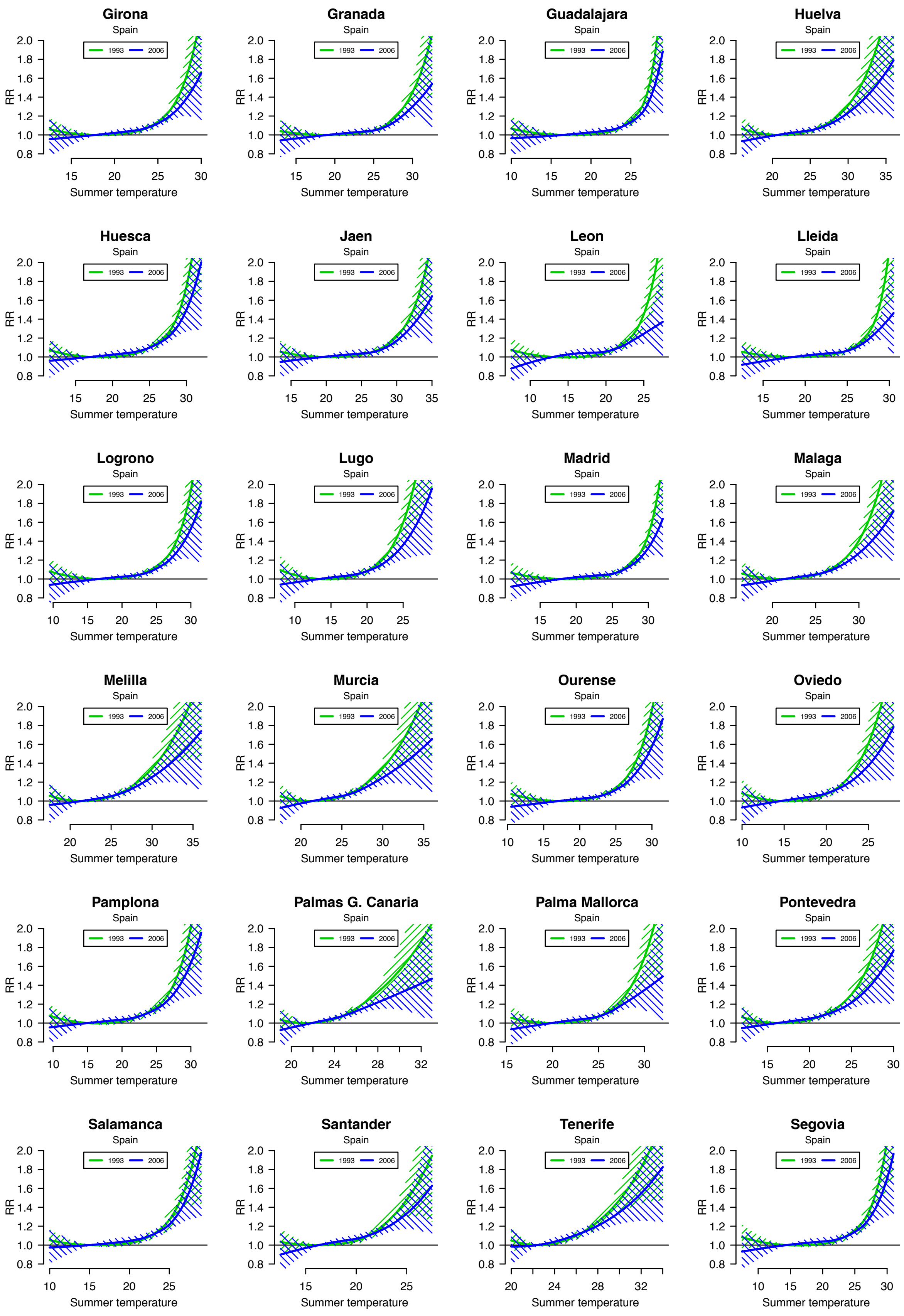
Girona
Spain
1993 2006
RR
Summer temperature
Granada
Spain
1993 2006
RR
Summer temperature
Guadalajara
Spain
1993 2006
RR
Summer temperature
Huelva
Spain
1993 2006
RR
Summer temperature
Huesca
Spain
1993 2006
RR
Summer temperature
Jaen
Spain
1993 2006
RR
Summer temperature
Leon
Spain
1993 2006
RR
Summer temperature
Lleida
Spain
1993 2006
RR
Summer temperature
Logrono
Spain
1993 2006
RR
Summer temperature
Lugo
Spain
1993 2006
RR
Summer temperature
Madrid
Spain
1993 2006
RR
Summer temperature
Malaga
Spain
1993 2006
RR
Summer temperature
Melilla
Spain
1993 2006
RR
Summer temperature
Murcia
Spain
1993 2006
RR
Summer temperature
Ourense
Spain
1993 2006
RR
Summer temperature
Oviedo
Spain
1993 2006
RR
Summer temperature
Pamplona
Spain
1993 2006
RR
Summer temperature
Palmas G. Canaria
Spain
1993 2006
RR
Summer temperature
Palma Mallorca
Spain
1993 2006
RR
Summer temperature
Pontevedra
Spain
1993 2006
RR
Summer temperature
Salamanca
Spain
1993 2006
RR
Summer temperature
Santander
Spain
1993 2006
RR
Summer temperature
Tenerife
Spain
1993 2006
RR
Summer temperature
Segovia
Spain
1993 2006
RR
Summer temperature

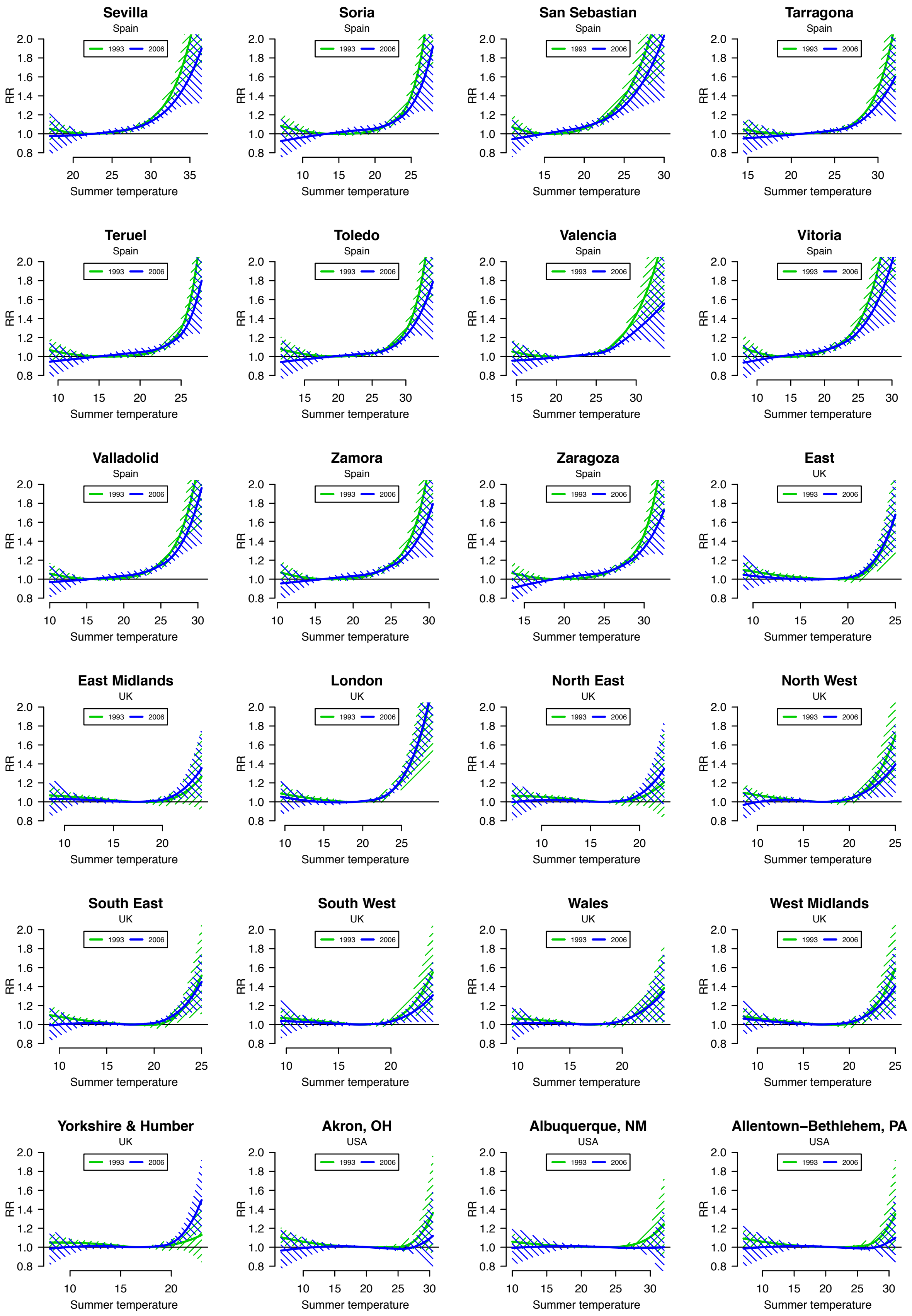
Sevilla
Spain
Soria
Spain
San Sebastian
Spain
Tarragona
Spain
Teruel
Spain
Toledo
Spain
Valencia
Spain
Vitoria
Spain
Valladolid
Spain
Zamora
Spain
Zaragoza
Spain
East
UK
East Midlands
UK
London
UK
North East
UK
North West
UK
South East
UK
South West
UK
Wales
UK
West Midlands
UK
Yorkshire & Humber
UK
Akron, OH
USA
Albuquerque, NM
USA
Allentown–Bethlehem, PA
USA
1993
2006
RR
Summer temperature

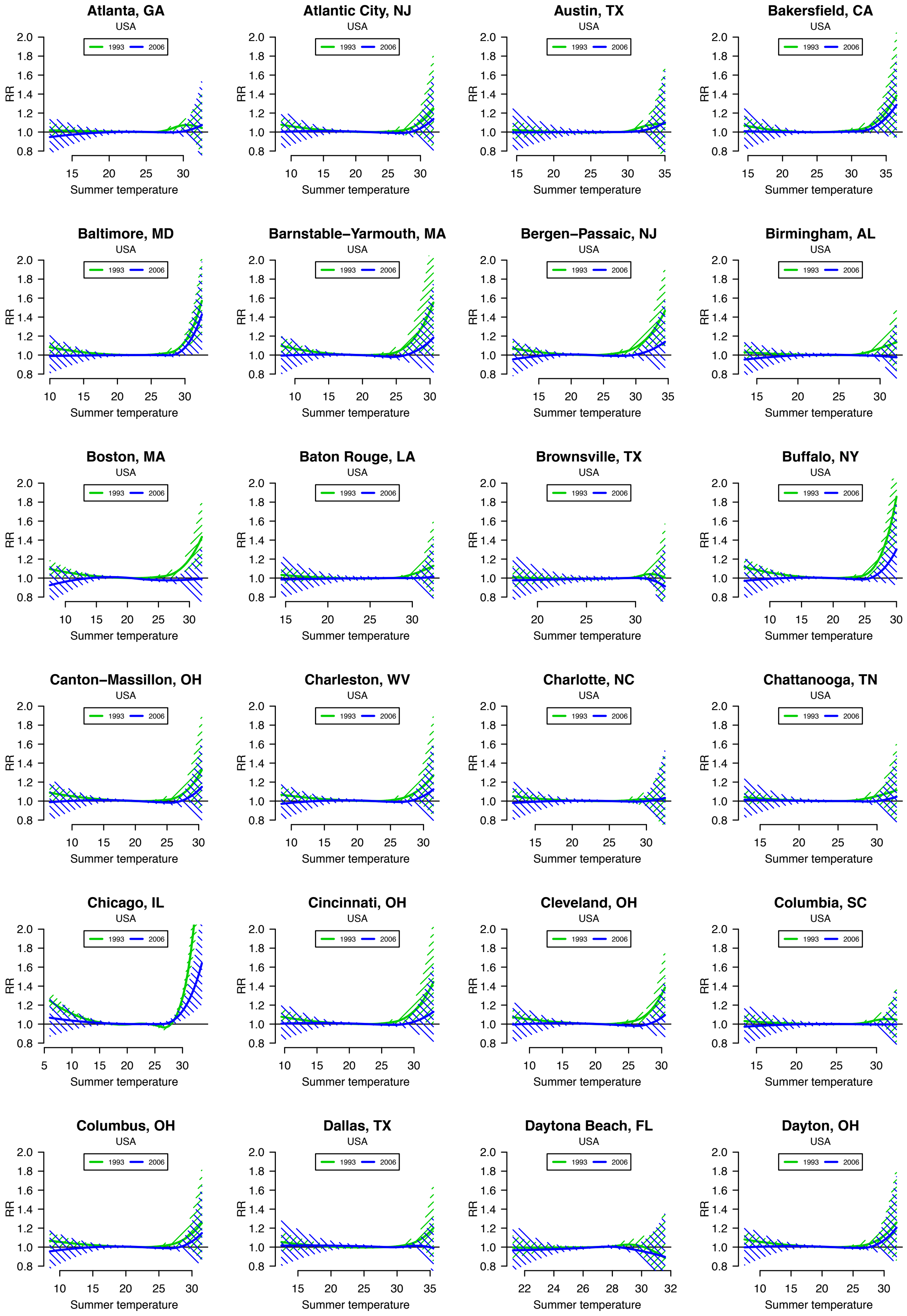
Atlanta, GA
USA
Atlantic City, NJ
USA
Austin, TX
USA
Bakersfield, CA
USA
Baltimore, MD
USA
Barnstable–Yarmouth, MA
USA
Bergen–Passaic, NJ
USA
Birmingham, AL
USA
Boston, MA
USA
Baton Rouge, LA
USA
Brownsville, TX
USA
Buffalo, NY
USA
Canton–Massillon, OH
USA
Charleston, WV
USA
Charlotte, NC
USA
Chattanooga, TN
USA
Chicago, IL
USA
Cincinnati, OH
USA
Cleveland, OH
USA
Columbia, SC
USA
Columbus, OH
USA
Dallas, TX
USA
Daytona Beach, FL
USA
Dayton, OH
USA
1993
2006
RR
Summer temperature

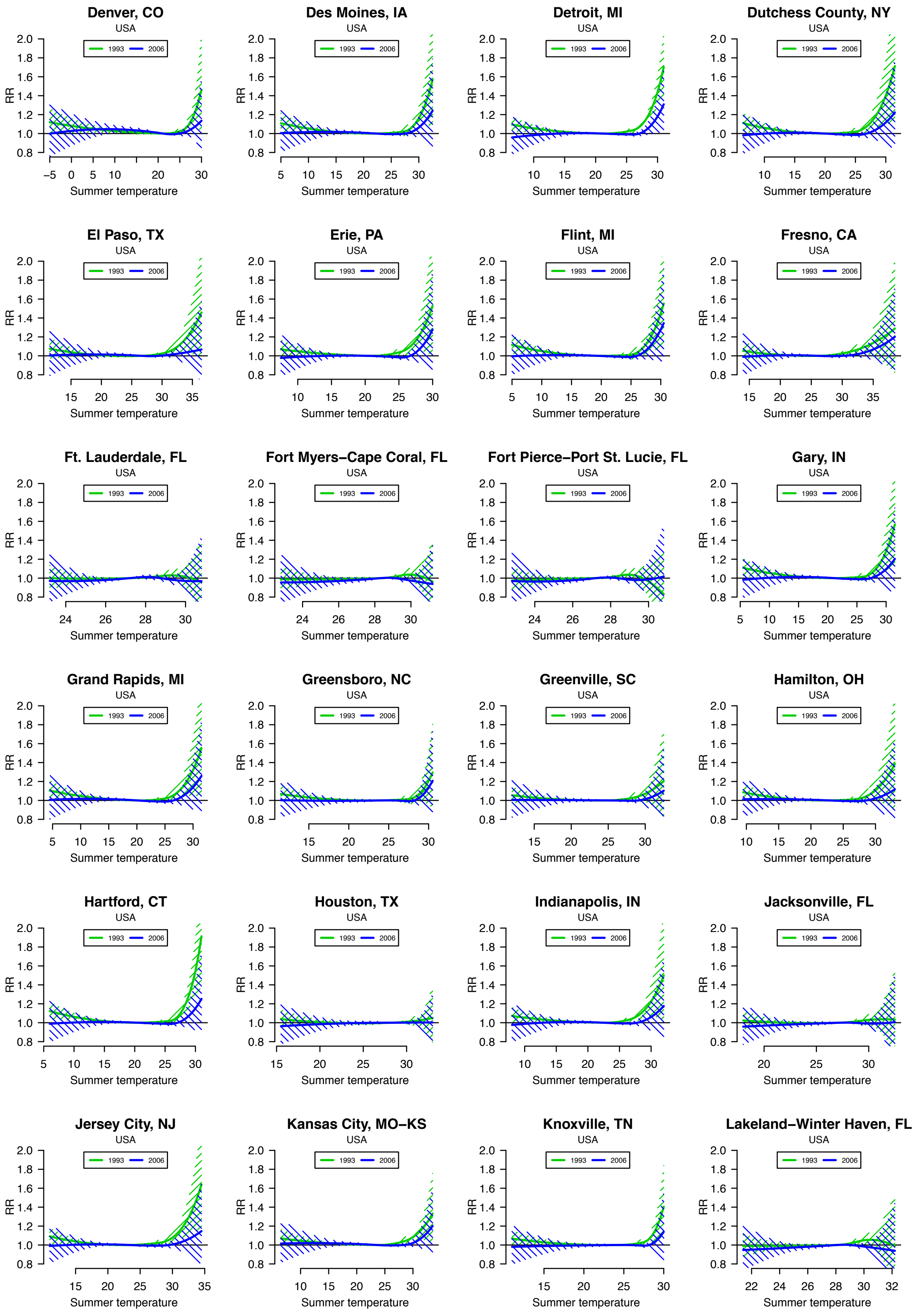
Denver, CO
USA
Des Moines, IA
USA
Detroit, MI
USA
Dutchess County, NY
USA
El Paso, TX
USA
Erie, PA
USA
Flint, MI
USA
Fresno, CA
USA
Ft. Lauderdale, FL
USA
Fort Myers–Cape Coral, FL
USA
Fort Pierce–Port St. Lucie, FL
USA
Gary, IN
USA
Grand Rapids, MI
USA
Greensboro, NC
USA
Greenville, SC
USA
Hamilton, OH
USA
Hartford, CT
USA
Houston, TX
USA
Indianapolis, IN
USA
Jacksonville, FL
USA
Jersey City, NJ
USA
Kansas City, MO–KS
USA
Knoxville, TN
USA
Lakeland–Winter Haven, FL
USA
1993
2006
RR
Summer temperature

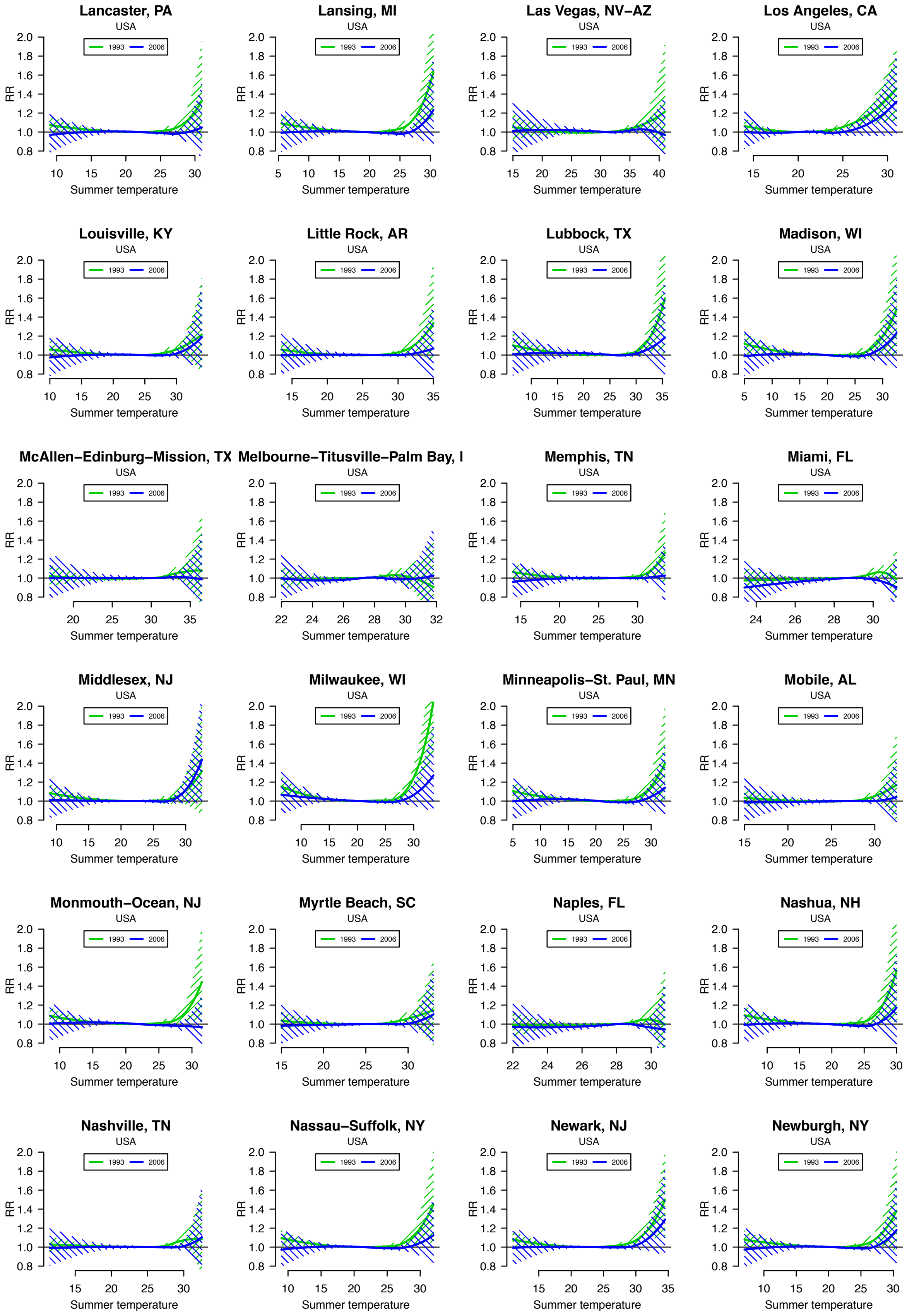
Lancaster, PA
USA
Lansing, MI
USA
Las Vegas, NV–AZ
USA
Los Angeles, CA
USA
Louisville, KY
USA
Little Rock, AR
USA
Lubbock, TX
USA
Madison, WI
USA
McAllen–Edinburg–Mission, TX
USA
Melbourne–Titusville–Palm Bay, I
USA
Memphis, TN
USA
Miami, FL
USA
Middlesex, NJ
USA
Milwaukee, WI
USA
Minneapolis–St. Paul, MN
USA
Mobile, AL
USA
Monmouth–Ocean, NJ
USA
Myrtle Beach, SC
USA
Naples, FL
USA
Nashua, NH
USA
Nashville, TN
USA
Nassau–Suffolk, NY
USA
Newark, NJ
USA
Newburgh, NY
USA
1993
2006
RR
Summer temperature

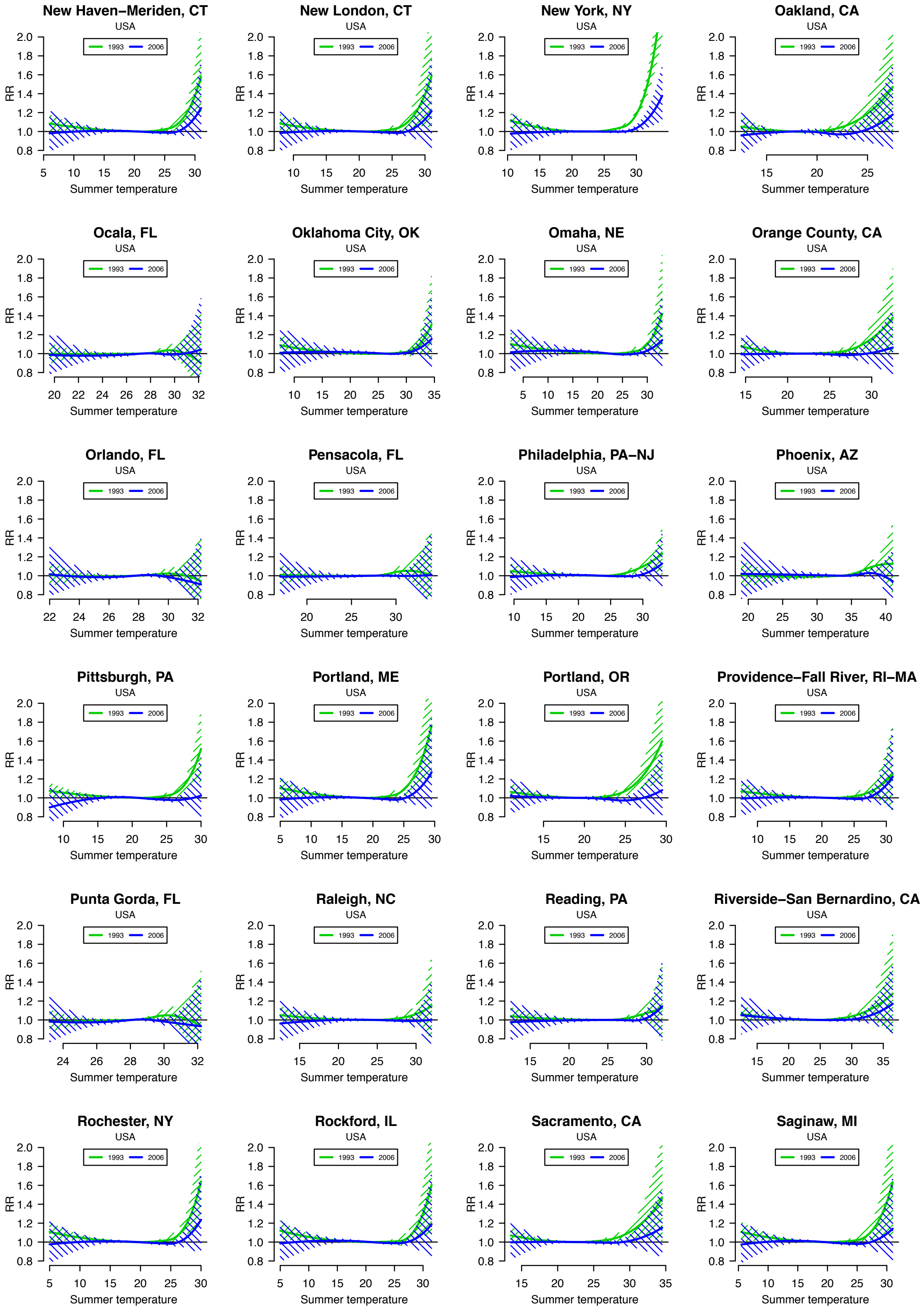
New Haven–Meriden, CT
USA
New London, CT
USA
New York, NY
USA
Oakland, CA
USA
Ocala, FL
USA
Oklahoma City, OK
USA
Omaha, NE
USA
Orange County, CA
USA
Orlando, FL
USA
Pensacola, FL
USA
Philadelphia, PA–NJ
USA
Phoenix, AZ
USA
Pittsburgh, PA
USA
Portland, ME
USA
Portland, OR
USA
Providence–Fall River, RI–MA
USA
Punta Gorda, FL
USA
Raleigh, NC
USA
Reading, PA
USA
Riverside–San Bernardino, CA
USA
Rochester, NY
USA
Rockford, IL
USA
Sacramento, CA
USA
Saginaw, MI
USA
1993
2006
RR
Summer temperature

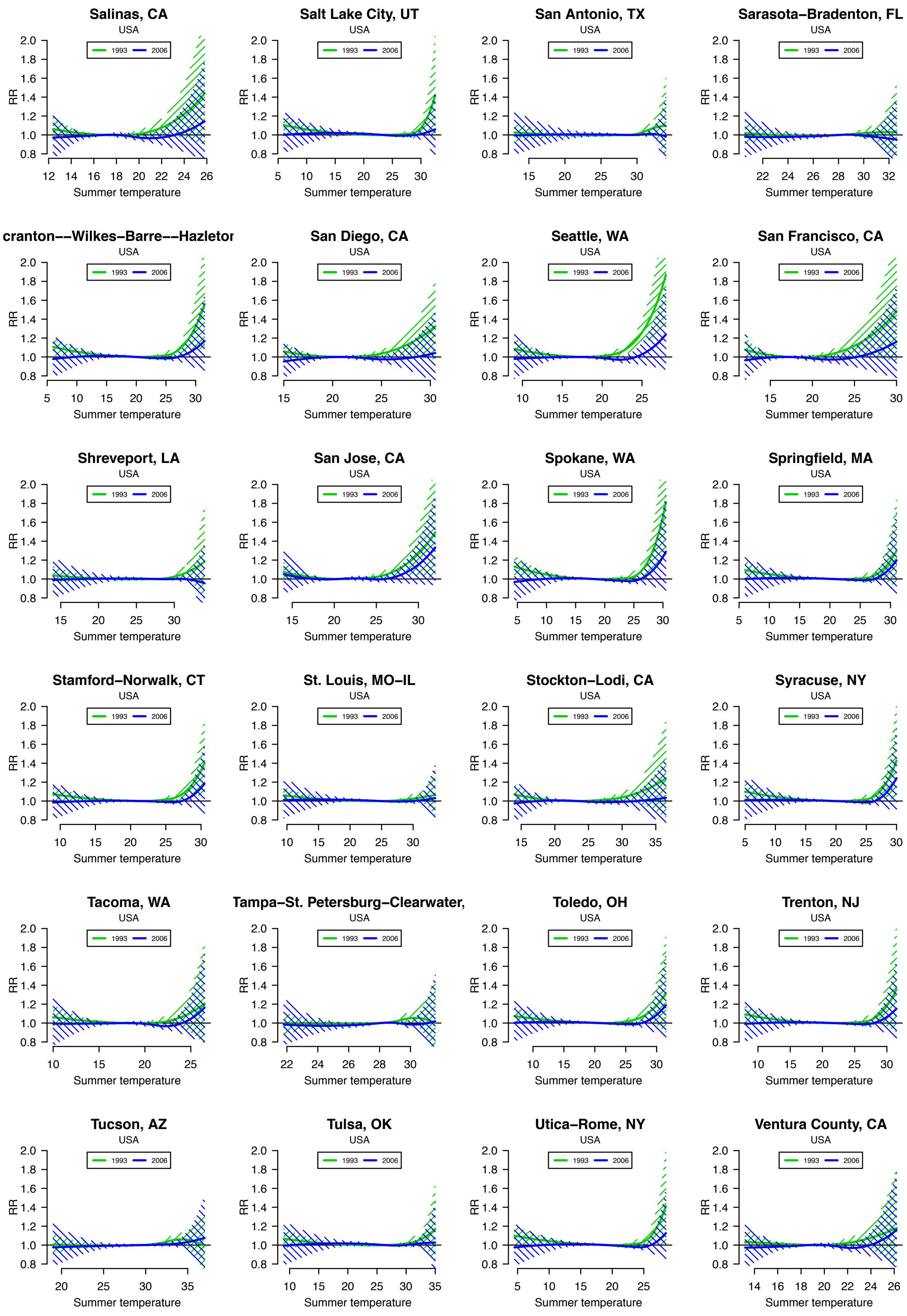
Salinas, CA
USA
Salt Lake City, UT
USA
San Antonio, TX
USA
Sarasota–Bradenton, FL
USA
cranton−−Wilkes−Barre−−Hazletor
USA
San Diego, CA
USA
Seattle, WA
USA
San Francisco, CA
USA
Shreveport, LA
USA
San Jose, CA
USA
Spokane, WA
USA
Springfield, MA
USA
Stamford–Norwalk, CT
USA
St. Louis, MO–IL
USA
Stockton–Lodi, CA
USA
Syracuse, NY
USA
Tacoma, WA
USA
Tampa–St. Petersburg–Clearwater,
USA
Toledo, OH
USA
Trenton, NJ
USA
Tucson, AZ
USA
Tulsa, OK
USA
Utica–Rome, NY
USA
Ventura County, CA
USA
1993
2006
RR
Summer temperature

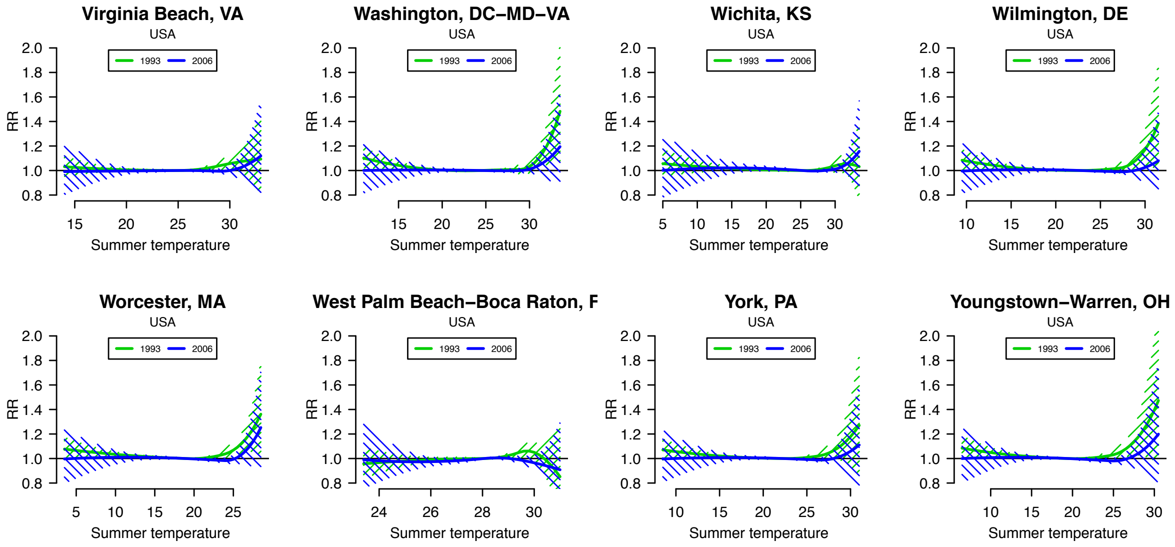
Virginia Beach, VA
USA
Washington, DC–MD–VA
USA
Wichita, KS
USA
Wilmington, DE
USA
Worcester, MA
USA
West Palm Beach–Boca Raton, F
USA
York, PA
USA
Youngstown–Warren, OH
USA
1993
2006
RR
Summer temperature

Figure S3. Effect modification of time on the overall cumulative exposure-response relationships[a] between heat and mortality in 7 countries, expressed as ratio of RR, with 95% confidence intervals. The vertical lines represent the minimum mortality temperature[b] (dotted) and the 90th and 99th percentiles of the temperature distribution (dashed). Note that the *y*-axis is scaled to the country-specific range. [a]The curves are represented on a relative scale of summer temperature percentiles, using country-specific distributions. [b]Estimated as the minimum of the overall cumulative exposure-response curve from the model without interaction (interpreted as the average across the whole study period).

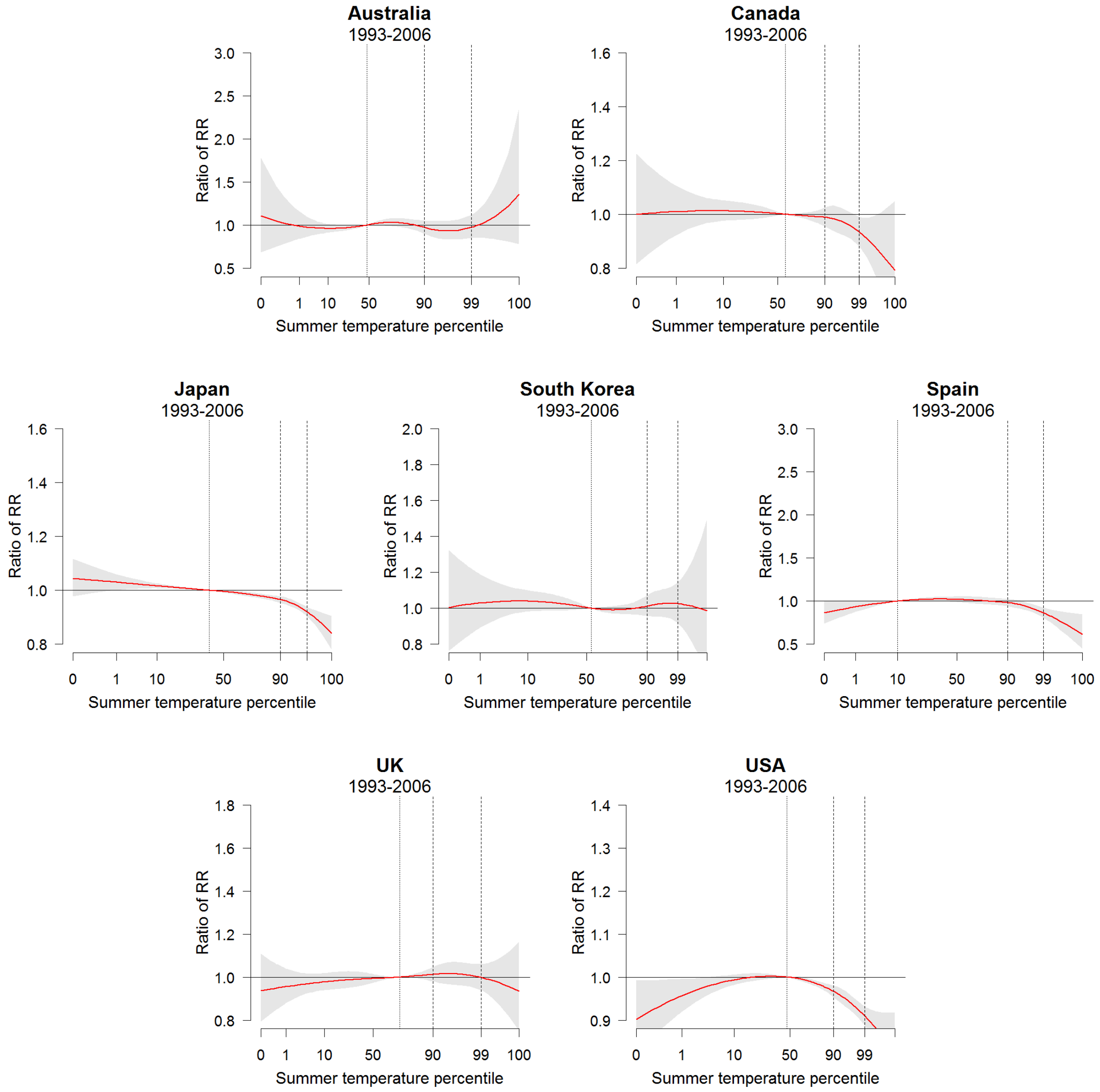

Figure S4. Lag-response relationships between heat and mortality predicted from the model with no interaction (interpreted as the average throughout the study period) in 7 countries, with 95% confidence intervals. These curves are computed for the temperature corresponding to the 99th percentile vs the country-specific minimum mortality temperature[a]. [a]Estimated as the minimum of the overall cumulative exposure-response curve from the model without interaction (interpreted as the average across the whole study period).

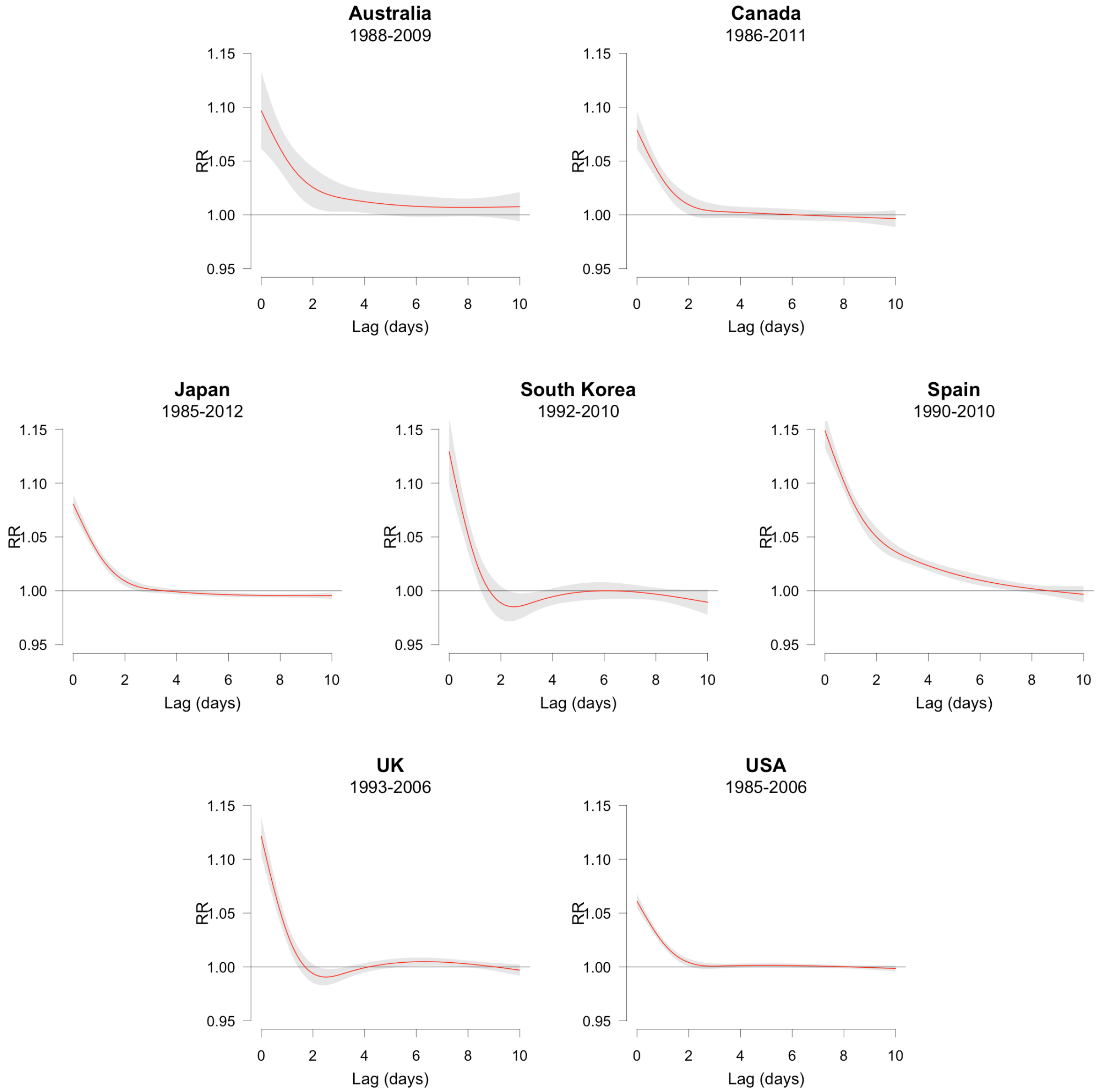

Figure S5. Corresponding to Figure 1 in the manuscript with maximum daily temperature as exposure index.[a] The vertical lines represent the minimum mortality temperature[b] (dotted) and the 90th and 99th percentiles of the temperature distribution (dashed). Note that the *y*-axis is scaled to the country-specific range. [a]The curves are represented on a relative scale of summer temperature percentiles, using country-specific distributions. [b]Estimated as the minimum of the overall cumulative exposure-response curve from the model without interaction (interpreted as the average across the whole study period).

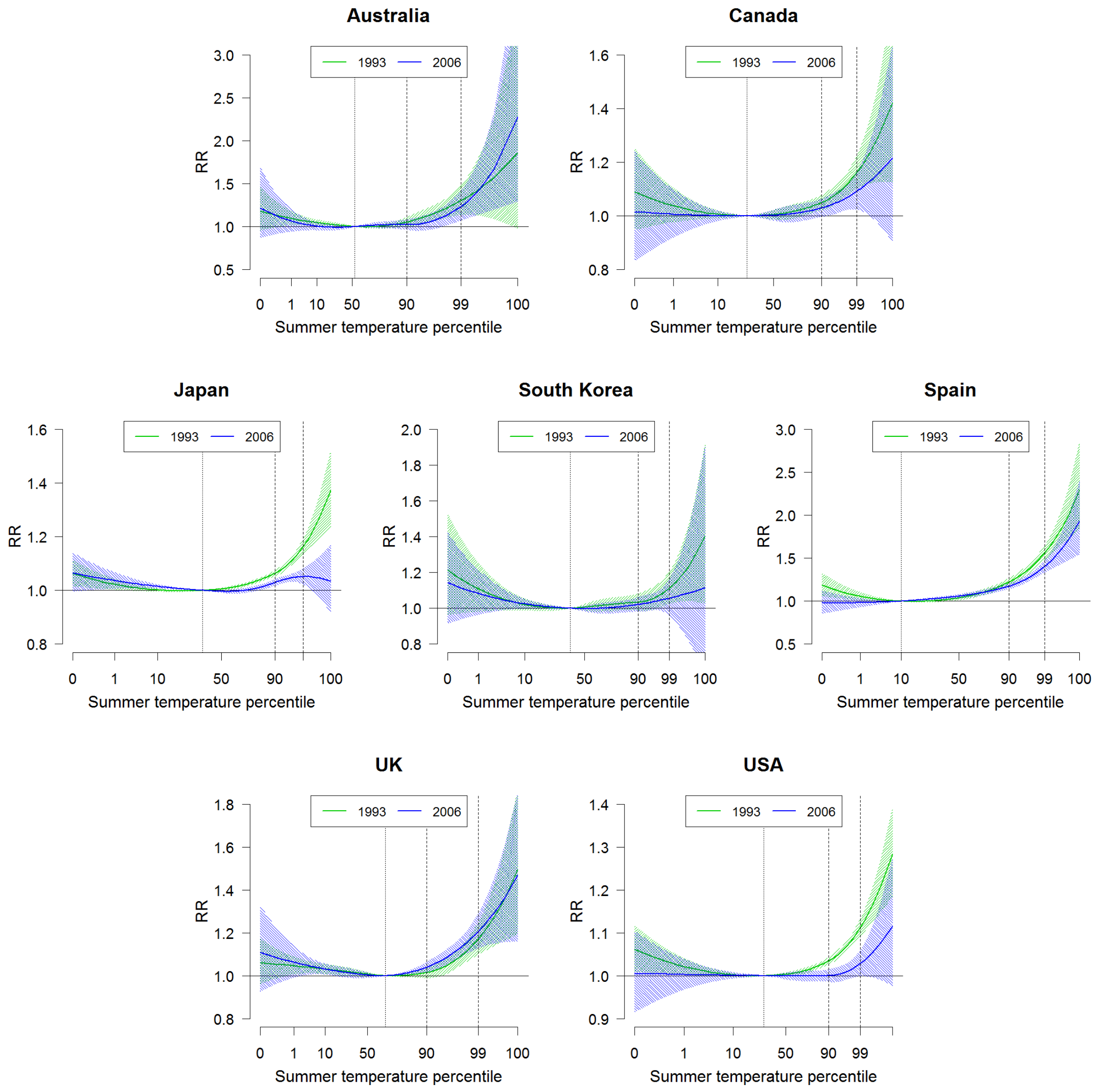

Figure S6. Corresponding to Figure 1 in the manuscript with minimum daily temperature as exposure index.[a] The vertical lines represent the minimum mortality temperature[b] (dotted) and the 90th and 99th percentiles of the temperature distribution (dashed). Note that the *y*-axis is scaled to the country-specific range. [a]The curves are represented on a relative scale of summer temperature percentiles, using country-specific distributions. [b]Estimated as the minimum of the overall cumulative exposure-response curve from the model without interaction (interpreted as the average across the whole study period).

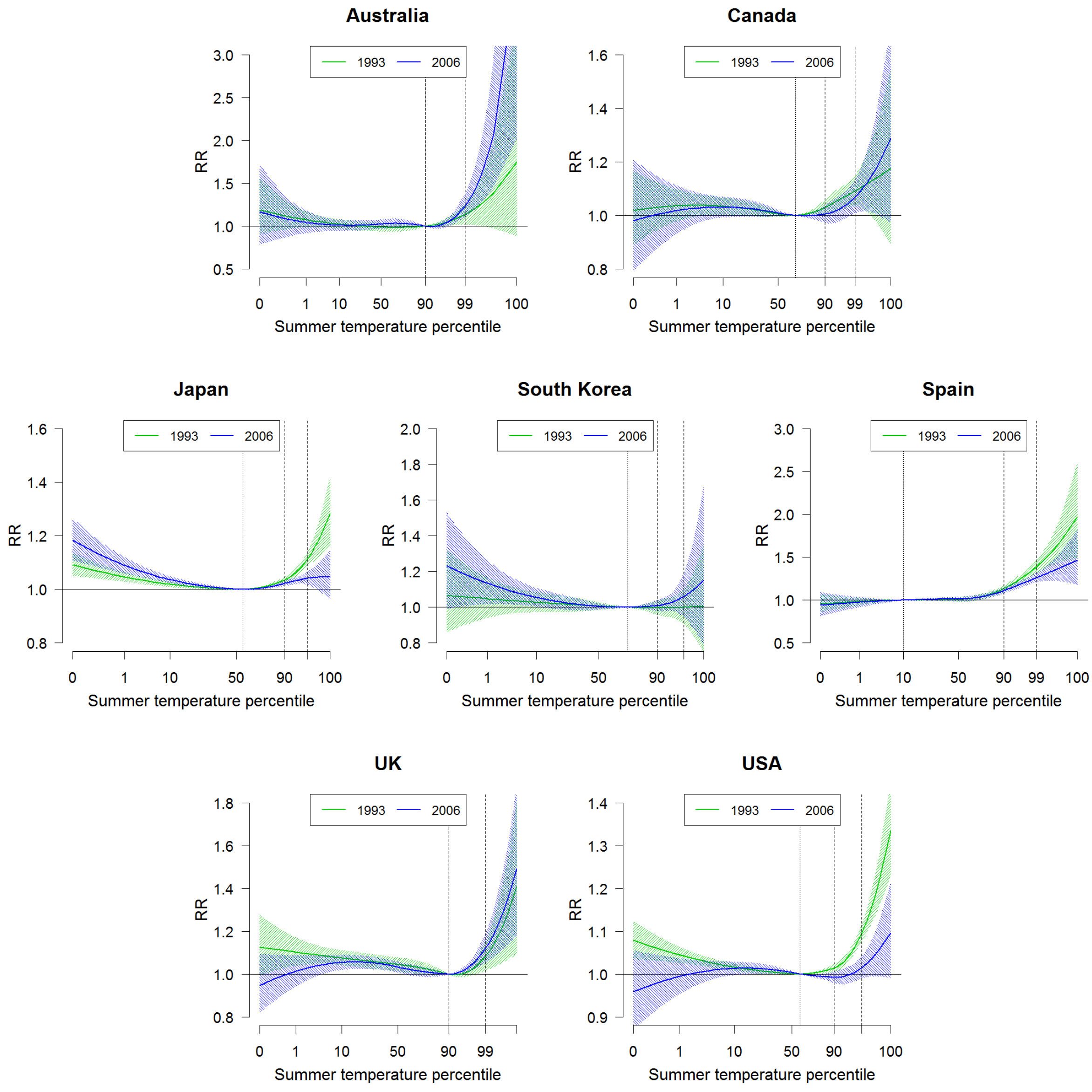

References

Armstrong BG, Chalabi Z, Fenn B, Hajat S, Kovats S, Milojevic A, et al. 2011. The association of mortality with high temperatures in a temperate climate: England and Wales. Journal of Epidemiology and Community Health 65:340-345.

Gasparrini A. 2011. Distributed lag linear and non-linear models in R: the package dlnm. Journal of Statistical Software 43:1-20.

Gasparrini A, Armstrong B, Kenward MG. 2012a. Multivariate meta-analysis for non-linear and other multi-parameter associations. Statistics in Medicine 31:3821-3839.

Gasparrini A, Armstrong B, Kovats S, Wilkinson P. 2012b. The effect of high temperatures on cause-specific mortality in England and Wales. Occupational and Environmental Medicine 69:56-61.

Kaplan GG, Tanyingoh D, Dixon E, Johnson M, Wheeler AJ, Myers RP, et al. 2013. Ambient ozone concentrations and the risk of perforated and nonperforated appendicitis: a multicity case-crossover study. Environmental Health Perspectives 121:939-943.

Martin SL, Cakmak S, Hebbern CA, Avramescu ML, Tremblay N. 2012. Climate change and future temperature-related mortality in 15 Canadian cities. International Journal of Biometeorology 56:605-619.

R Core Team. 2014. R: A Language and Environment for Statistical Computing. Vienna, Austria:R Foundation for Statistical Computing.

Tobias A, Armstrong B, Zuza I, Gasparrini A, Linares C, Diaz J. 2012. Mortality on extreme heat days using official thresholds in Spain: a multi-city time series analysis. BMC Public Health 12:133.

Tong S, Wang XY, Guo Y. 2012. Assessing the short-term effects of heatwaves on mortality and morbidity in Brisbane, Australia: comparison of case-crossover and time series analyses. PloS One 7:e37500.

Tong S, Wang XY, Yu W, Chen D, Wang X. 2014. The impact of heatwaves on mortality in Australia: a multicity study. BMJ Open 4:e003579.

Zanobetti A, Schwartz J. 2009. The effect of fine and coarse particulate air pollution on mortality: a national analysis. Environmental Health Perspectives 117:898-903.

Zanobetti A, O'Neill MS, Gronlund CJ, Schwartz JD. 2013. Susceptibility to mortality in weather extremes: effect modification by personal and small-area characteristics. Epidemiology 24:809-819.
